# Activation of PKA/SIRT1 signaling pathway by photobiomodulation therapy reduces Aβ levels in Alzheimer's disease models

**DOI:** 10.1111/acel.13054

**Published:** 2019-10-30

**Authors:** Zhan Zhang, Qi Shen, Xiaolei Wu, Di Zhang, Da Xing

**Affiliations:** ^1^ MOE Key Laboratory of Laser Life Science & Institute of Laser Life Science South China Normal University Guangzhou China; ^2^ College of Biophotonics South China Normal University Guangzhou China

**Keywords:** Alzheimer's disease, amyloid‐β, APP processing, cAMP/PKA pathway, photobiomodulation therapy, SIRT1

## Abstract

A hallmark of Alzheimer's disease (AD) is the accumulation of amyloid‐β (Aβ), which correlates significantly with progressive cognitive deficits. Although photobiomodulation therapy (PBMT), as a novel noninvasive physiotherapy strategy, has been proposed to improve neuronal survival, decrease neuron loss, ameliorate dendritic atrophy, and provide overall AD improvement, it remains unknown whether and how this neuroprotective process affects Aβ levels. Here, we report that PBMT reduced Aβ production and plaque formation by shifting amyloid precursor protein (APP) processing toward the nonamyloidogenic pathway, thereby improving memory and cognitive ability in a mouse model of AD. More importantly, a pivotal protein, SIRT1, was involved in this process by specifically up‐regulating ADAM10 and down‐regulating BACE1, which is dependent on the cAMP/PKA pathway in APP/PS1 primary neurons and SH‐SY5Y cells stably expressing human APP Swedish mutation (APPswe). We further found that the activity of the mitochondrial photoacceptor cytochrome c oxidase (CcO) was responsible for PBMT‐induced activation of PKA and SIRT1. Together, our research suggests that PBMT as a viable therapeutic strategy has great potential value in improving cognitive ability and combatting AD.

## Graphical abstract

Aβ overproduction plays a pivotal role in the pathogenesis of AD. Herein, we firstly revealed that photobiomodulation therapy (PBMT) as a drug‐free and noninvasive physiotherapy strategy was capable of lowering Aβ levels by shifting APP processing toward the nonamyloidogenic pathway, which is dependent on SIRT1 deacetylase activity. Photoactivation of mitochondrial photoacceptor cytochrome c oxidase (CcO) is responsible for the activation of PKA/SIRT1 signaling pathway.

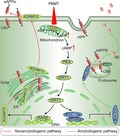

## 1 INTRODUCTION

Alzheimer's disease (AD), the most common irreversible and degenerative brain disease causing dementia in the elderly, is characterized by progressive memory loss and cognitive deterioration (Selkoe, 2001). Clinically, pathological features of AD are extracellular senile plaques composed of fibrillar amyloid‐β (Aβ) peptides and intracellular neurofibrillary tangles containing hyperphosphorylated tau, accompanied by synaptic dysfunction and neuronal death (Mangialasche, Solomon, Winblad, Mecocci, & Kivipelto, 2010). Despite copious research, the mechanisms governing this disease remain elusive.

Currently, the Aβ cascade hypothesis continues to dominate AD research, with abundant evidence supporting that Aβ overproduction alters cellular metabolism, triggers downstream events including tau hyperphosphorylation, neurodegeneration, neuroinflammation, and synaptic dysfunction, and further causes progressive memory loss and cognitive dysfunction (Billings, Oddo, Green, McGaugh, & LaFerla, 2005; Hardy & Selkoe, 2002). Aβ peptides are derived from the sequential proteolytic processing of the amyloid precursor protein (APP) by β‐secretase (β‐site APP cleaving enzyme 1, BACE1) and γ‐secretase consisting of three major components: presenilin 1 (PS1), nicastrin, and Pen‐2, with this cleavage step contributing heavily to AD pathology (O'Brien & Wong, 2011). However, Aβ production can be avoided through a nonamyloidogenic pathway by α‐secretase (mainly a disintegrin and metalloproteinase domain‐containing protein 10, ADAM10) followed by the γ‐secretase (Lichtenthaler & Haass, 2004). Therefore, inhibition of Aβ production by regulating APP processing may be an important strategy to attenuate cognitive deficits during AD pathology.

Sirtuins are a family of NAD^+^‐dependent protein deacetylases known to have beneficial effects against age‐related diseases, such as cancer, diabetes, cardiovascular, and neurodegenerative diseases (Guarente, 2006). Of the seven mammalian sirtuins, the SIR2 ortholog Sirtuin1 (SIRT1) plays an essential role in improving neurodegenerative diseases by influencing neuron survival, neurite outgrowth, synaptic plasticity, cognitive function, and neurogenesis (Herskovits & Guarente, 2014). SIRT1 has been proposed to induce protective effects against AD pathology through regulating the acetylation homeostasis of key proteins (Donmez, 2012; Herskovits & Guarente, 2014). Importantly, SIRT1 is highly expressed in neurons of the hippocampus, an important region for learning and memory that is vulnerable to AD (Mu & Gage, 2011). These results imply that activating SIRT1 may be an important approach to protect against AD.

Photobiomodulation therapy (PBMT), as a novel, drug‐free, and noninvasive physiotherapy strategy, has shown to regulate neuronal functions in cell cultures, animal models, and clinical conditions (Eells et al., 2004; Rojas, Lee, John, & Gonzalez‐Lima, 2008). Previous studies have shown that the application of PBMT could provide effective neuroprotection from Aβ‐induced neuronal cell apoptosis, decrease neuronal loss, and ameliorate dendrite atrophy (Liang, Liu, & Xing, 2012; Meng, He, & Xing, 2013; Zhang, Wu, & Xing, 2012). PBMT can also efficiently and noninvasively penetrate into biologic tissue including the CNS, producing beneficial photobiomodulation effects, such as promoting nerve regeneration and increasing ATP synthesis (Anders, Borke, Woolery, & Merwe, 1993; Mochizuki‐Oda et al., 2002). There is evidence suggesting that a primary mitochondrial chromophore for photobiostimulation is cytochrome c oxidase (CcO), which has been suggested to be the key photoacceptor of light in the far red to near‐IR spectral range (Eells et al., 2004; Pastore, Greco, & Passarella, 2000). In addition to an increase in ATP formation, photobiostimulation may also result in a cascade of signaling events (Karu, Pyatibrat, Kolyakov, & Afanasyeva, 2005). Such properties support the notion that PBMT may have great potential for the treatment of neurodegeneration.

In this study, we revealed that PBMT is capable of lowering Aβ levels and amyloid plaque burden by shifting APP processing toward the nonamyloidogenic pathway, which is dependent on the SIRT1 deacetylase activity. We report, for the first time, that PBMT activates SIRT1 via the cAMP/PKA pathway. Importantly, the current study found that the mitochondrial photoacceptor CcO is responsible for PBMT‐induced activation of PKA and SIRT1. Thus, these findings provide further evidence of photobiomodulation which may provide a novel therapeutic strategy to control the progression of AD by targeting Aβ production.

## 2 RESULTS

### 2.1 PBMT reduces Aβ levels in APP/PS1 transgenic mice

**Figure 1:**
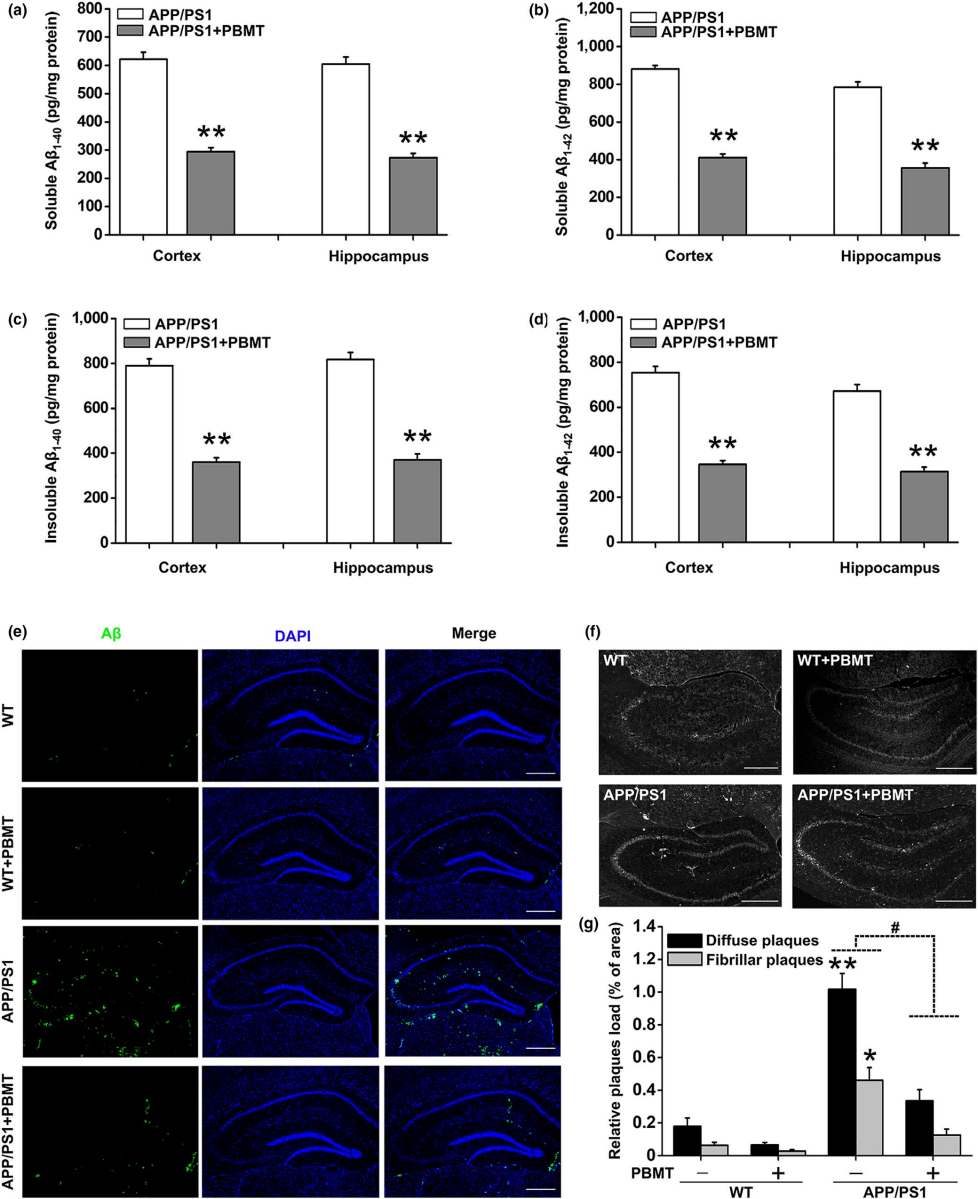
PBMT reduces cerebral Aβ levels and amyloid plaque burden in APP/PS1 transgenic mice. (a–d) Cerebral cortex and hippocampus Aβ measurements were performed by ELISA. APP/PS1 mice (*n* = 7) under PBMT treatment showed dramatic reductions in the levels of soluble Aβ_1‐40_ (a) and Aβ_1‐42_ (b), insoluble Aβ_1‐40_ (c) and Aβ_1‐42_ (d) compared with control transgenic mice (*n* = 7). (e) Representative images immunostained with Aβ‐specific 6E10 antibody in the brain of PBMT‐treated WT and APP/PS1 mice (*n* = 5). Scale bar: 300 μm. (f) Representative images of thioflavin T‐stained senile plaques in the brains of different groups of mice (*n* = 5). Scale bar: 300 μm. (g) The values in the bar graph are expressed in the percentage area occupied by diffuse plaques and fibrillar plaques. All the data are reported as mean ± *SEM*. *p < 0.05 and **p < 0.01 versus the control group; #*p* < 0.05 versus the indicated group

Transcranial PBMT has shown promising effects on the treatment of stroke, traumatic brain injury, and neurodegenerative disease (Naeser & Hamblin, 2011). Our previous findings indicated that PBMT significantly improves neuronal functions (Meng et al., 2013; Zhang et al., 2012). However, amyloid plaque burden and cognitive function under PBMT have not been evaluated. To establish the physiological relevance of amyloid modulation by PBMT in AD, we employed a mouse model in which two linked transgenes, encoding the human APPswe and PSEN1dE9 (henceforth referred to as APP/PS1) alleles, drive Aβ production and deposition in the brain with increasing age. The heads of the six‐month‐old male mice were shaved (without removing the scalp and skull) and treated with PBMT above the head for 10 min/day (approximately 2 J/cm^2^ reaching the interior of the hippocampus) for 30 days and then analyzed at 7 months of age. First, Aβ levels were detected by enzyme‐linked immunosorbent assay (ELISA) in both the cerebral cortex and hippocampus of APP/PS1 mice. Biochemical analysis revealed a dramatic reduction in soluble Aβ_1−40_ and Aβ_1−42_ in APP/PS1 mice after treatment with PBMT (Figure 1a,b). Moreover, PBMT also produced a marked decrease in the levels of insoluble Aβ_1−40_ and Aβ_1−42_ (Figure 1c,d).

To further determine the effects of PBMT on Aβ deposits, we used Aβ‐specific 6E10 antibody to detect diffuse plaques (Figure 1e) and thioflavin T to detect fibrillar plaques (Figure 1f). Consistent with the effect of PBMT on Aβ accumulation, pathological evaluation of AD‐relevant brain areas in APP/PS1 mice revealed that mice accumulated numerous amyloid plaques throughout the brain, while the staining of diffuse plaques and fibrillar plaques showed markedly weaker immunoreactivity following PBMT treatment. Quantitative analysis revealed that long‐term exposure to PBMT significantly reduced the percentage area occupied by diffuse plaques and fibrillar plaques (Figure 1g). Overall, these results suggest that PBMT effectively reduces Aβ levels and amyloid plaque burden in vivo.

### 2.2 PBMT rescues spatial learning and memory deficits in APP/PS1 mice

**Figure 2:**
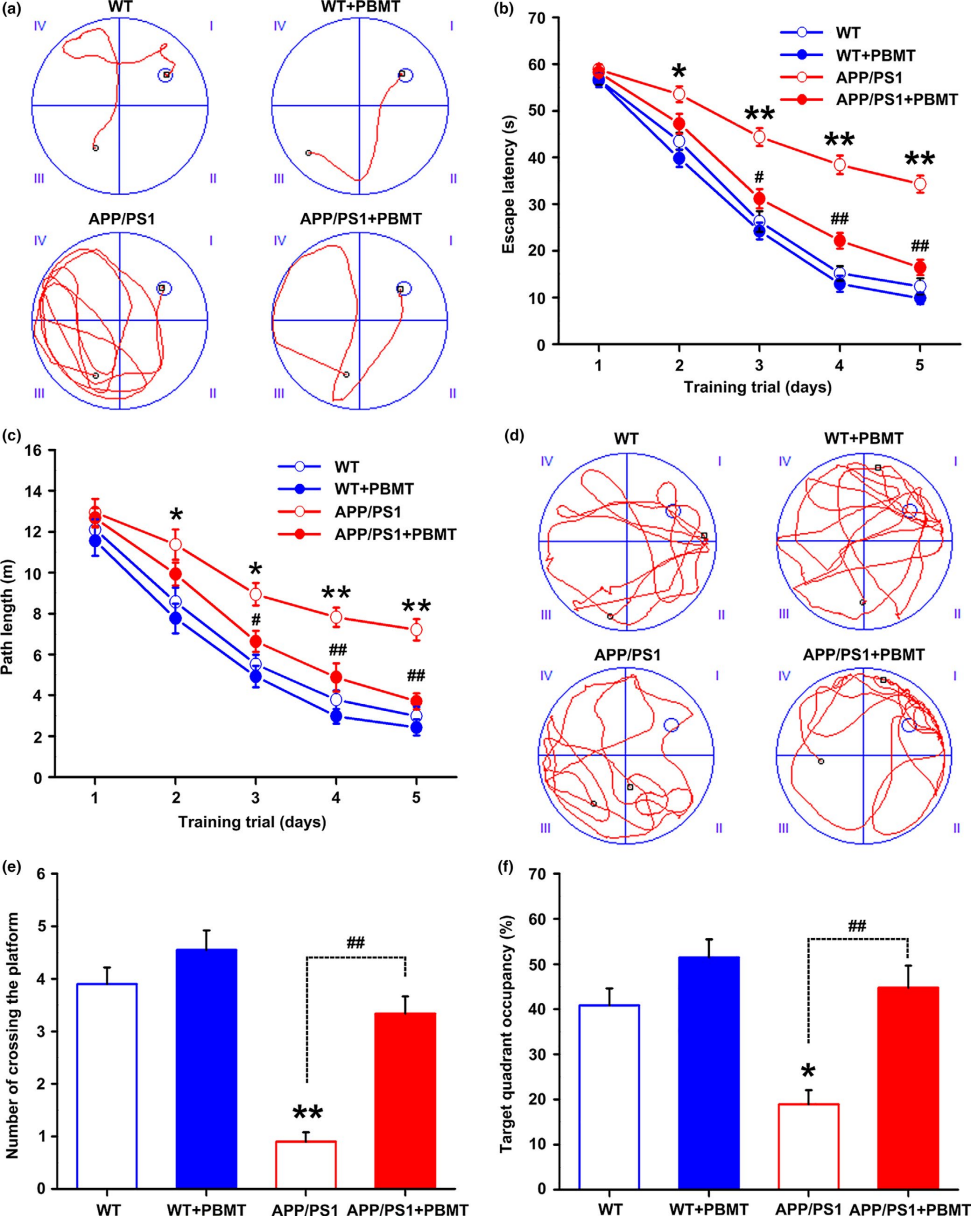
PBMT improves spatial learning and memory of APP/PS1 mice in the MWM. (a) Representative swimming traces on day 5 during the place navigation trial showing the effects of PBMT on the spatial memory of WT and APP/PS1 mice. (b) The escape latency of mice to find the hidden platform was recorded on each training day. (c) Mean distance per day for path length was shown during acquisition of the hidden platform task. (d) Representative swimming traces of the four groups of mice exhibited after the training trial. (e) The number of crossing the platform during a 60‐s probe trial of MWM test. (f) Percentage of time spent in target quadrant of the original platform position. All the data are reported as mean ± *SEM*. *n* = 9 animals per group. *p < 0.05 and **p < 0.01 versus the WT group; #*p* < 0.05 and ##*p* < 0.01 versus the APP/PS1 group

One of the most major factors for the development of AD is Aβ overproduction in the brain, which correlates well with spatial learning and memory dysfunction in APP/PS1 mice (Billings et al., 2005; Trinchese et al., 2004). To investigate the effects of PBMT on Aβ‐induced behavioral deficits, the spatial learning and memory of mice were evaluated by using the Morris water maze (MWM). In the visible platform test, there was no group difference in swimming strategies (Figure S1a). Subsequently, spatial learning was assessed by the time required to find the hidden platform (escape latency) across the 5‐day acquisition training period. In the acquisition phase, representative swimming paths on the 5th day of place navigation trials displayed clear differences among groups (Figure 2a). Mice from all groups showed a day‐to‐day decline in escape latency during training, and this decline was more significant as the training days progressed in the PBMT group (Figure 2b). However, the average swimming speed did not differ significantly among the groups in this trial (Figure S1b). In addition, the path length to find the platform was longer for APP/PS1 mice than PBMT‐treated APP/PS1 mice (Figure 2c), which indicated that PBMT substantially improved learning deficits. APP/PS1 mice tended to swim everywhere in the spatial probe test, whereas mice in other groups preferred to move in the platform quadrant performed on the 6th day (Figure 2d). Similarly, mice in PBMT‐treated group crossed the former platform location more often and spent more time in the target quadrant than those in the nontreated APP/PS1 group (Figure 2e,f). These results indicate that PBMT effectively rescues spatial learning and memory deficits in APP/PS1 mice.

### 2.3 PBMT alters APP processing to promote the nonamyloidogenic pathway by up‐regulating ADAM10 and down‐regulating BACE1, both in vivo and in vitro

**Figure 3:**
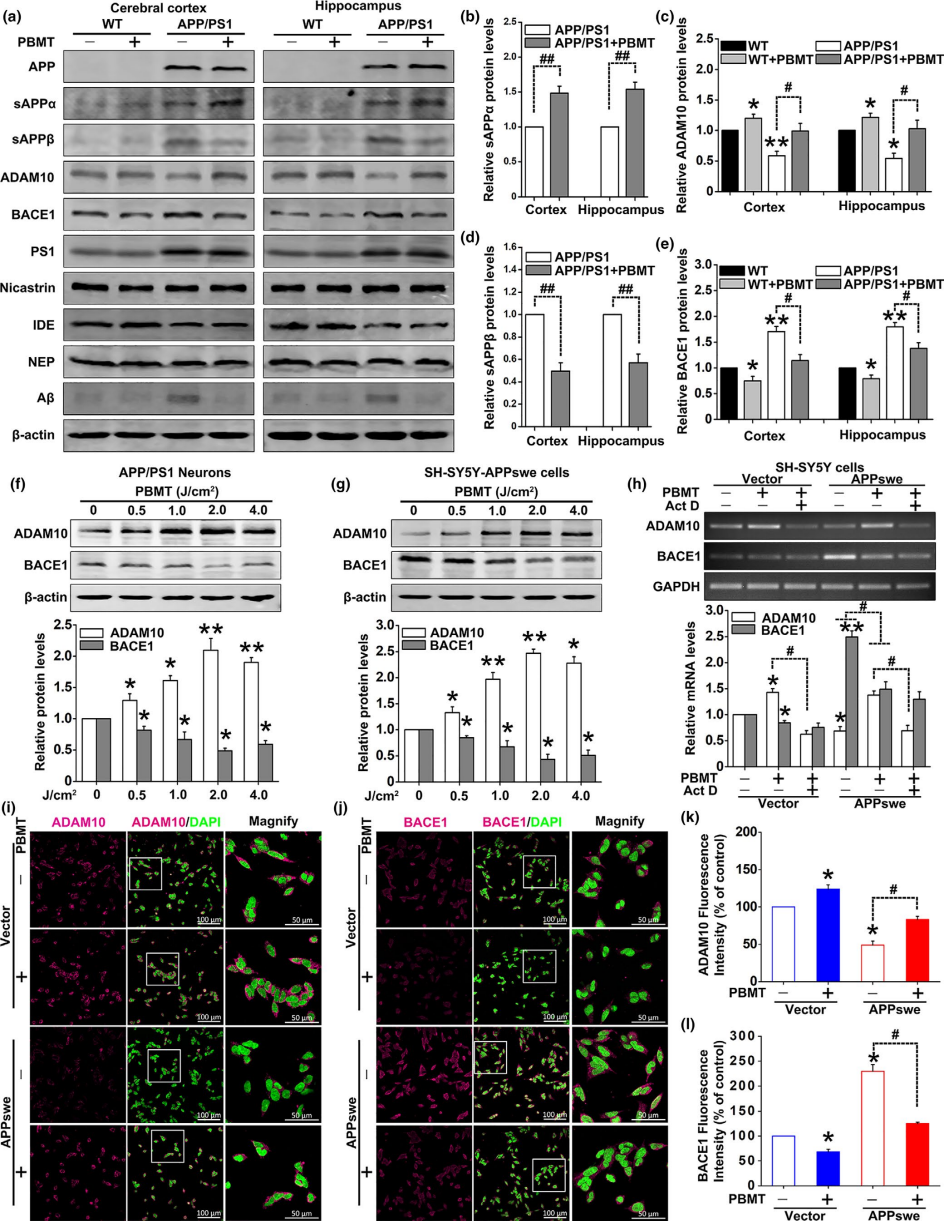
Effects of PBMT on APP processing in vivo and in vitro. (a) Representative Western blot bands of Aβ, APP, sAPPα, sAPPβ, ADAM10, BACE1, PS1, nicastrin, IDE, and NEP in the cerebral cortex and hippocampus of WT and APP/PS1 mice (*n* = 5), whether or not with PBMT. (b–e) Densitometric quantification of exogenous human sAPPα (b) and sAPPβ (d), endogenous mouse ADAM10 (c) and BACE1 (e) expressions after indicated treatments. (f, g) Representative Western blot assays for detecting the dose‐dependent effects of PBMT on endogenous ADAM10 and BACE1 expressions in APP/PS1 neurons (f) and SH‐SY5Y‐APPswe cells (g). (h) ADAM10 and BACE1 mRNA levels were detected by PCR stimulated with PBMT in the presence of Act D (10 μM) in SH‐SY5Y cells. (i, j) Representative immunofluorescent images of ADAM10 (i) and BACE1 (j) in SH‐SY5Y cells. Staining with DAPI to visualize nucleus. (k, l) The fluorescence intensity data of ADAM10 (k) and BACE1 (l) were recorded by confocal microscopy. All the data are reported as mean ± *SEM* of four independent experiments. *p < 0.05 and **p < 0.01 versus the control group; #*p* < 0.05 and ##*p* < 0.01 versus the indicated group

We next explored the underlying mechanisms of the reduction in Aβ protein levels by PBMT. It has been previously reported that the expression of APP is positively correlated with Aβ generation in AD (O'Brien & Wong, 2011). We found that Aβ levels markedly decreased in PBMT‐treated APP/PS1 mice, but no significant differences were observed in the levels of full‐length APP between nontreated and PBMT‐treated APP/PS1 mice (Figure 3a; Figure S2a,b). Additionally, we also found no differences in the protein levels of IDE and NEP, the main proteolytic enzymes responsible for cerebral Aβ degradation, between the two groups of APP/PS1 mice, regardless of whether they were treated with PBMT (Figure S2c,d).

Abnormally increased Aβ production is closely correlated with APP processing. Therefore, we assessed the levels of major APP‐cleaved products sAPPα and sAPPβ. Quantitative analysis showed that PBMT markedly increased the level of sAPPα and decreased the level of sAPPβ fragments (Figure 3b,d). Two key enzymes, ADAM10 and BACE1, are involved in the initial cleavage of APP toward sAPPα and sAPPβ formation (O'Brien & Wong, 2011). Thus, we further analyzed the expressions of ADAM10 and BACE1. Notably, PBMT could blunt the down‐regulation of ADAM10 and significantly down‐regulate BACE1 protein levels in APP/PS1 mice (Figure 3c,e). Additionally, there were no significant differences in the protein levels of PS1 and nicastrin, the major components of γ‐secretase, between nontreated and PBMT‐treated APP/PS1 mice (Figure S2e,f). These data indicate that, in an AD mouse model, the lowering of Aβ levels by PBMT is attributable to changes in APP processing toward the nonamyloidogenic pathway.

To further confirm the effects of PBMT on ADAM10 and BACE1 expressions, we employed two AD model cells, primary hippocampal neurons derived from APP/PS1 mice (APP/PS1 neurons) and SH‐SY5Y cells stably expressing the APPswe transgene (SH‐SY5Y‐APPswe cells). Western blot analysis of the cell lysate samples demonstrated dramatic reductions in baseline levels of ADAM10, as well as increases in APP and BACE1 protein levels in APP/PS1 primary neurons (Figure S3a). Similar changes were also observed in SH‐SY5Y‐APPswe cells (Figure S3b).

Our previous studies demonstrated that PBMT with 2 J/cm^2^ could improve neuronal functions (Meng et al., 2013; Zhang et al., 2012). Thus, we began treatment with 2 J/cm^2^ PBMT for research in vitro. Our results showed that this dose of laser significantly increased ADAM10 expression and decreased BACE1 expression in both APP/PS1 neurons and SH‐SY5Y‐APPswe cells (Figure S3c,d). Additionally, PBMT regulated ADAM10 and BACE1 expressions in a dose‐dependent manner, with statistically significant increases in ADAM10 levels and decreases in BACE1 levels observed at the dose of 0.5, 1, 2, and 4 J/cm^2^ (Figure 3f,g). The dose of 2 J/cm^2^ PBMT was selected as the subject of study in the following experiments. Immunofluorescence staining also showed that fluorescence intensities of ADAM10 were increased, and BACE1 were decreased in SH‐SY5Y‐APPswe cells with PBMT (Figure 3i–l). In addition, we investigated whether the PBMT‐induced increase in ADAM10 protein required new mRNA synthesis by pharmacologically blocking transcription with Act D. We observed that pretreatment of SH‐SY5Y‐APPswe cells with Act D significantly inhibited the PBMT‐induced increase in ADAM10 mRNA expression (Figure 3h). Meanwhile, we found that mRNA expression of BACE1 was also significantly decreased in PBMT‐treated cells, which was consistent with the experimental results of protein levels.

### 2.4 Activating SIRT1 is responsible for PBMT‐induced up‐regulation of ADAM10 and down‐regulation of BACE1

**Figure 4:**
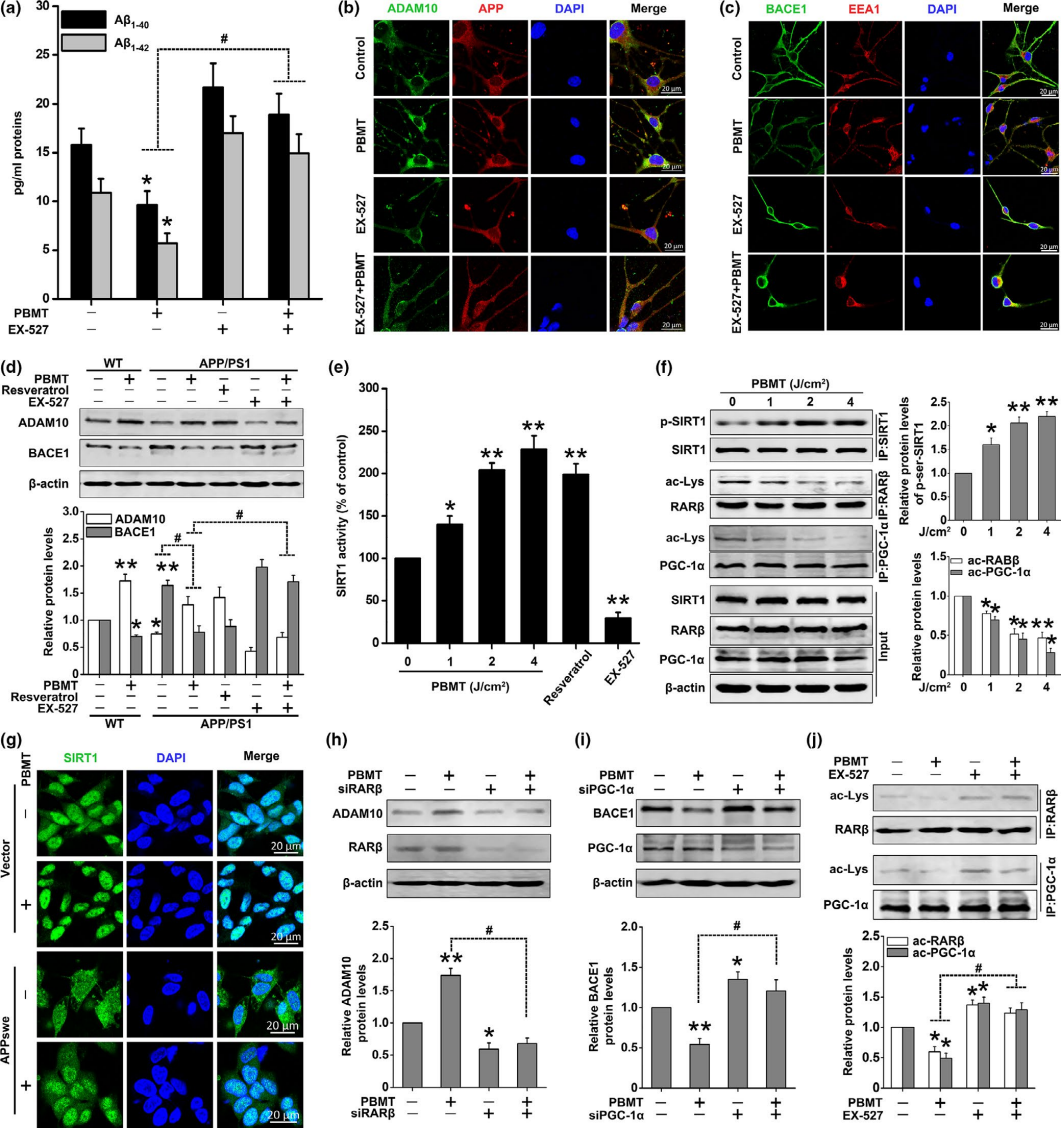
PBMT‐induced SIRT1‐coupled RARβ and PGC‐1α deacetylations are responsible for ADAM10 and BACE1 expressions, respectively. (a) EX‐527 (20 μM) blocked the effects of PBMT in which Aβ_1‐40_ and Aβ_1‐42_ released into the culture media are reduced, in APP/PS1 neurons. (b) Representative immunofluorescent images of ADAM10 in APP/PS1 neurons. Staining with DAPI to visualize the nucleus. (c) Representative immunofluorescent images of BACE1 in APP/PS1 neurons. Staining with EEA1 antibody to visualize endosome. (d) Western blot analysis of ADAM10 and BACE1 expressions after treatment with PBMT in the presence of EX‐527 (20 μM) and resveratrol (20 μM) in primary neurons. (e) Relative SIRT1 deacetylase activity for detecting the dose‐dependent effects of PBMT in APP/PS1 neurons. EX‐527 was used as a negative control, and resveratrol was used as a positive control. (f) Immunoprecipitates were analyzed for detecting the dose‐dependent effects of PBMT on p‐SIRT1, ac‐RARβ, and ac‐PGC‐1α levels in SH‐SY5Y‐APPswe cells. (g) Representative immunofluorescent images of SIRT1 in SH‐SY5Y cells. (h, i) Western blot analysis of ADAM10 expression after treatment with RARβ siRNA (h) and BACE1 expression after treatment with PGC‐1α siRNA (i) in PBMT‐treated SH‐SY5Y‐APPswe cells. (j) Immunoprecipitates were analyzed for detecting ac‐RARβ and ac‐PGC‐1α levels after treatment with EX‐527 in PBMT‐treated SH‐SY5Y‐APPswe cells. All the data are reported as mean ± *SEM* of four independent experiments. *p < 0.05 and **p < 0.01 versus the control group; #*p* < 0.05 versus the indicated group

Several studies had demonstrated that SIRT1 activity participates in the expressions of ADAM10 and BACE1, ultimately inhibiting Aβ production (Qin et al., 2006; Shah et al., 2017; Wang et al., 2013). We observed that pretreatment of APP/PS1 neurons with EX‐527 (a specific inhibitor of SIRT1) significantly inhibited PBMT‐induced decreases in Aβ_1‐40_ and Aβ_1‐42_ secretion (Figure 4a). EX‐527 significantly blocked the effects of PBMT on ADAM10 and BACE1 expressions in APP/PS1 neurons (Figure 4d). Concomitantly, we also found that EX‐527 significantly inhibited the effects of PBMT on ADAM10 and BACE1 mRNA levels in SH‐SY5Y‐APPswe cells (Figure S4a). Further, SH‐SY5Y‐APPswe cells were transfected with SIRT1 siRNA. Knocking down SIRT1 antagonized the PBMT‐induced up‐regulation of ADAM10 and down‐regulation of BACE1 (Figure S4b). These results were also confirmed by immunofluorescent staining with antibodies against ADAM10 or BACE1 in APP/PS1 primary neurons (Figure 4b,c; Figure S4c,d).

To test whether PBMT could enhance SIRT1 deacetylase activity, an SIRT1 activity assay was performed in APP/PS1 neurons. We found that PBMT enhanced the deacetylase activity of SIRT1 in a dose‐dependent manner (Figure 4e). It has been previously suggested that post‐translational phosphorylated modifications to SIRT1 increase deacetylase activity of SIRT1 (Gerhart‐Hines et al., 2011). Meanwhile, the phosphorylation level of SIRT1 was increased in SH‐SY5Y‐APPswe cells after treatment with PBMT (Figure 4f, Figure S5a).

Since SIRT1 is a nuclear–cytoplasm shuttle protein (Tanno, Sakamoto, Miura, Shimamoto, & Horio, 2007), we wanted to determine whether PBMT could promote SIRT1 into the nucleus and then deacetylate the related transcription factors. We observed that SIRTI levels in the nucleus increased after PBMT in SH‐SY5Y‐APPswe cells (Figure S5b). Similar results were obtained by immunocytochemistry staining with antibodies against SIRT1 (Figure 4g). Protein localization of SIRT1 was primarily located in the nucleus of SH‐SY5Y control cells, while some SIRT1 shuttled from the nucleus to the cytoplasm in SH‐SY5Y‐APPswe cells. In summary, these findings suggest that PBMT modulates the transcription and expression of ADAM10 and BACE1 by activating SIRT1 and promoting its translocation to the nucleus.

### 2.5 SIRT1‐coupled RARβ and PGC‐1α deacetylation is necessary for PBMT to up‐regulate ADAM10 and down‐regulate BACE1

We next investigated the relevant transcription factors involved in PBMT‐mediated expressions of ADAM10 and BACE1. It has been reported that the activation of SIRT1‐coupled RARβ attenuates Aβ production in vitro, via the up‐regulation of ADAM10, suggesting a protective role for SIRT1 in AD (Lee et al., 2014). Additionally, activation of SIRT1 could also decrease BACE1 activity and inhibit Aβ production by promoting PGC‐1α activity in neurons (Wang et al., 2013). To confirm whether PBMT could deacetylate the relative transcription factors of ADAM10 and BACE1, we first detected the acetylation levels of RARβ and PGC‐1α in vivo. We found that the acetylation levels of RARβ and PGC‐1α in the cerebral cortex of APP/PS1 mice were decreased by PBMT (Figure S6). Similarly, we also observed PBMT‐induced decreases in acetylation levels of RARβ and PGC‐1α in a dose‐dependent manner in SH‐SY5Y‐APPswe cells (Figure 4f). Further, knocking down RARβ antagonized the PBMT‐induced up‐regulation of ADAM10 (Figure 4h). Meanwhile, we silenced PGC‐1α protein using its siRNA. As shown in Figure 4i, PBMT‐induced decrease in BACE1 expression was markedly attenuated. In addition, we found that pretreatment of SH‐SY5Y‐APPswe cells with EX‐527 significantly inhibited PBMT‐induced decreases in acetylation levels of RARβ and PGC‐1α (Figure 4j). All of these data support the idea that PBMT up‐regulates ADAM10 through a SIRT1‐coupled RARβ‐dependent transcription and down‐regulates BACE1 through a SIRT1‐coupled PGC‐1α‐dependent transcription.

### 2.6 PBMT activates SIRT1 via the cAMP/PKA pathway

**Figure 5:**
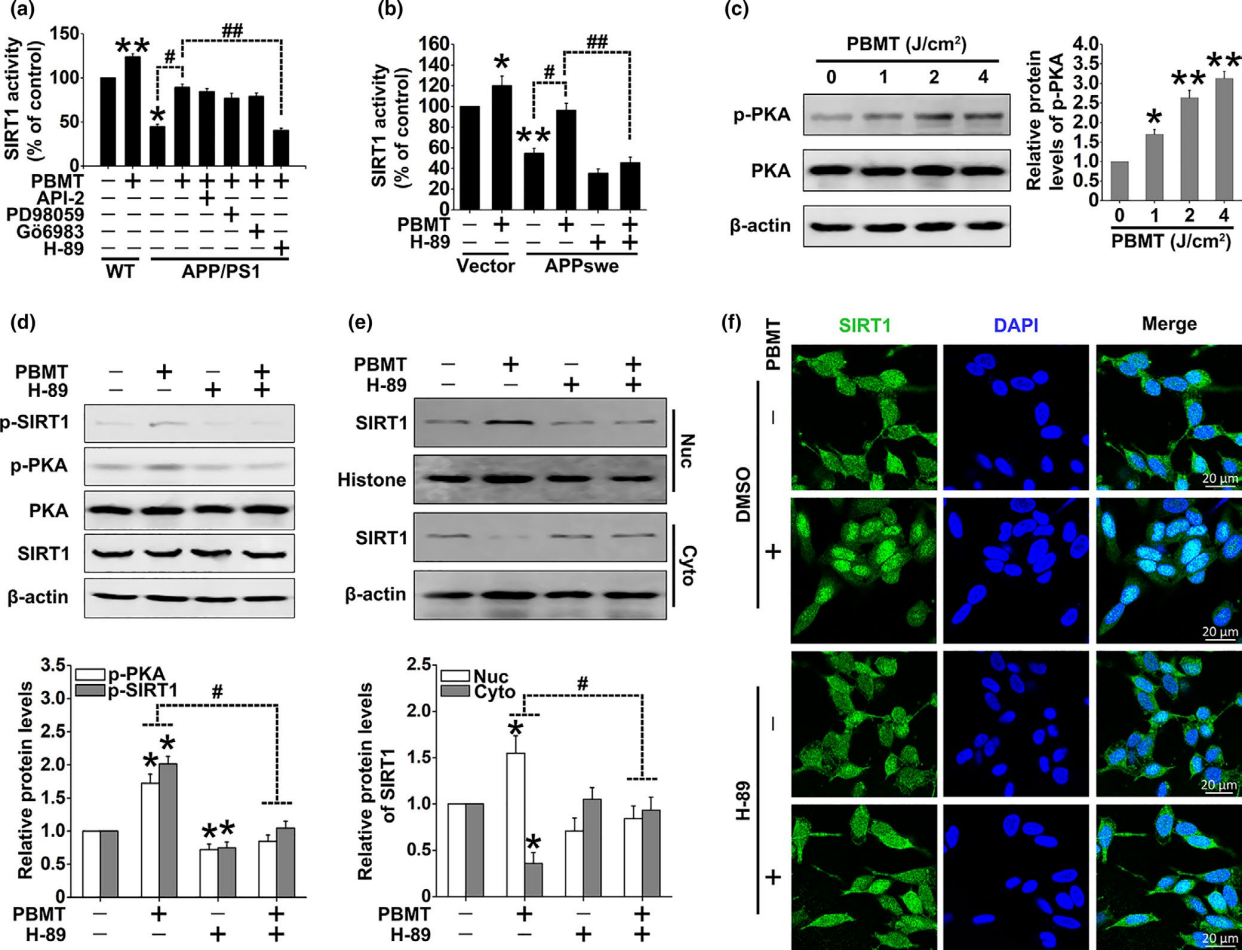
PBMT enhances SIRT1 activity via the cAMP/PKA pathway. (a) SIRT1 activity assay kit was used to detect SIRT1 deacetylase activity after treatment with PBMT in the presence of API‐2 (2 μM), PD98059 (1 μM), Gӧ6983 (20 μM), and H‐89 (20 μM) in neurons. (b) SIRT1 deacetylase activity was detected after the indicated treatments in SH‐SY5Y cells. (c) Representative Western blot assay for detecting the dose‐dependent effects of PBMT on p‐PKA in APP/PS1 neurons. (d) Representative Western blot assay for detecting the levels of p‐PKA and p‐SIRT1 after treatment with H‐89 in SH‐SY5Y‐APPswe cells. (e) Representative Western blot assay for detecting the levels of SIRT1 after indicated treatments in cytoplasm (Cyto) and nuclear (Nuc) lysates of SH‐SY5Y‐APPswe cells, respectively. (f) Representative immunofluorescent images of SIRT1 (green) in SH‐SY5Y‐APPswe cells under the indicated treatments. Staining with DAPI (blue) to visualize nucleus. All the data are reported as mean ± *SEM* of four independent experiments. *p < 0.05 and **p < 0.01 versus the control group; #*p* < 0.05 and ##*p* < 0.01 versus the indicated group

To investigate the possible kinases activated by PBMT responsible for the SIRT1 activation, we studied the effects of specific inhibitors of these pathways: API‐2, PD98059, Gӧ6983, and H‐89 (for Akt, MEK/ERKs, PKCs, and PKA, respectively) in SIRT1 deacetylase activity assays. We found that PBMT increased SIRT1 deacetylase activity in APP/PS1 neurons, which was reversed by pretreatment with H‐89, but not API‐2, PD98059, or Gӧ6983 (Figure 5a). To further confirm whether PKA was involved in PBMT‐induced SIRT1 deacetylase activation, we found the same effect in SH‐SY5Y‐APPswe cells (Figure 5b). Importantly, PBMT‐induced PKA phosphorylation was observed in a dose–response manner in APP/PS1 neurons (Figure 5c). Western blot assays were further performed to examine the effects of PKA activation on SIRT1 phosphorylation in response to PBMT in SH‐SY5Y‐APPswe cells. As shown in Figure 5d, increases in p‐PKA (activated PKA) and p‐SIRT1 (activated SIRT1) were seen in the samples after PBMT treatment. Inhibition of PKA by H‐89 before PBMT resulted in decreased phosphorylation of SIRT1. We also found that SIRT1 levels in the nucleus increased after PBMT (Figure 5e,f). However, after the cells were pre‐incubated with H‐89, PBMT markedly prevented SIRT1 from entering the nucleus. These results indicate that PBMT‐induced SIRT1 activation is in a cAMP/PKA‐dependent pathway.

### 2.7 PBMT‐induced PKA/SIRT1 activation depends on activity of the mitochondrial photoacceptor CcO

**Figure 6:**
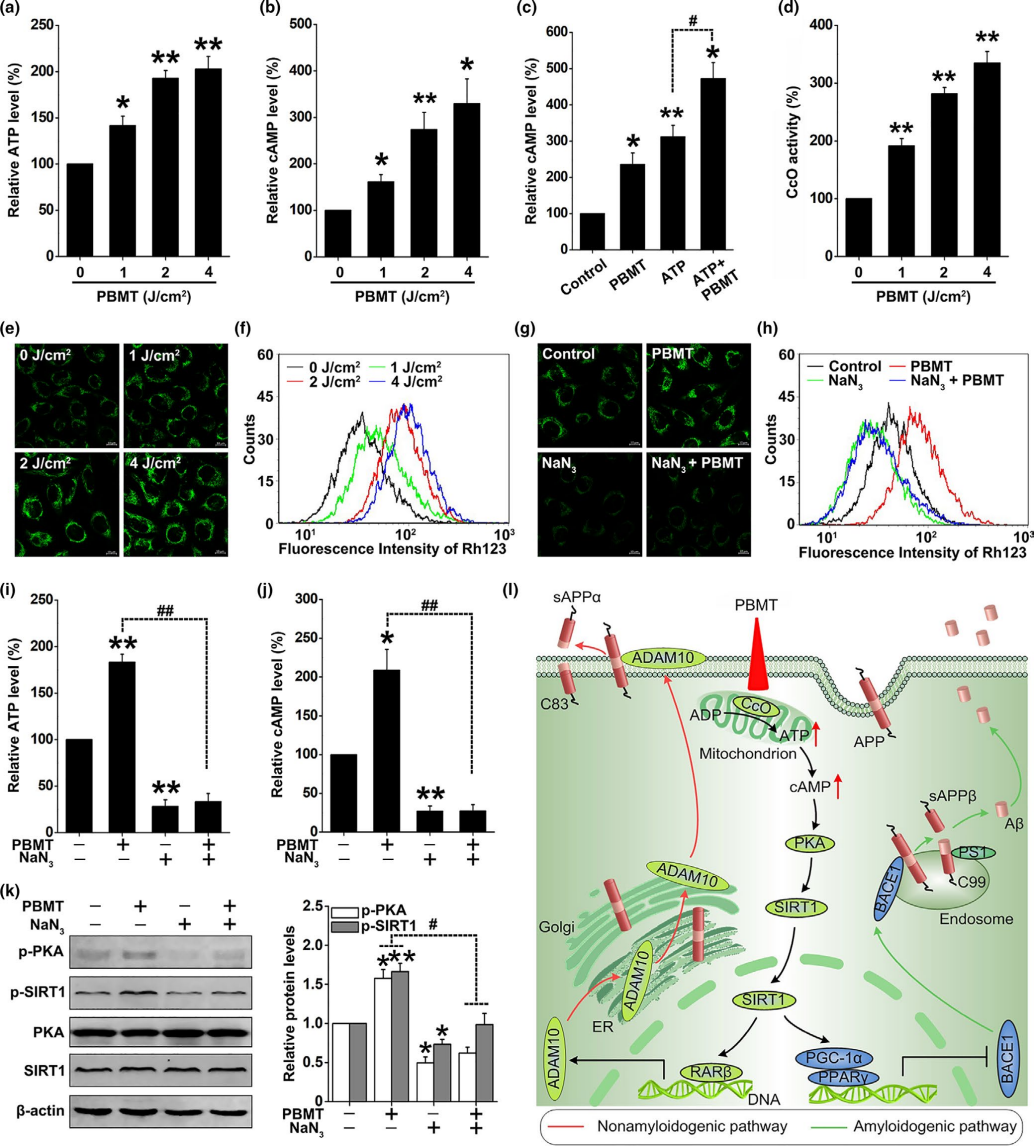
PBMT enhances mitochondrial photoacceptor CcO activity, increases ATP and cAMP levels, and further activates PKA/SIRT1 pathway in SH‐SY5Y‐APPswe cells. (a, b) SH‐SY5Y‐APPswe cells were treated with 0, 1, 2, and 4 J/cm^2^ PBMT. Relative ATP (a) and cAMP (b) levels were calculated as the percentage of the 0 J/cm^2^ dose level. (c) Relative cAMP content of SH‐SY5Y‐APPswe cells was determined after PBMT (2 J/cm^2^), with or without ATP (100 μM). (d) The measurement of CcO activity was determined in SH‐SY5Y‐APPswe cells under 0, 1, 2, and 4 J/cm^2^ PBMT. (e, f) The mitochondrial membrane potential (ΔΨmt) was detected by confocal microscopy (e) and flow cytometry (f) in Rhodamine 123 (Rh123) labeling SH‐SY5Y‐APPswe cells with indicated treatments. (g, h) ΔΨmt was detected by confocal microscopy (g) and flow cytometry (h) in Rh123 labeling SH‐SY5Y‐APPswe cells after treatment with NaN_3_ (10 mM). (i, j) SH‐SY5Y‐APPswe cells were treated with NaN_3_. Relative ATP (i) and cAMP (j) levels were calculated as the percentage of the control group level. (k) Western bolt analysis of p‐PKA and p‐SIRT1 levels after treatment with NaN_3_ in PBMT‐treated SH‐SY5Y‐APPswe cells. (l) Schematic representation of the signaling pathway for PBMT reducing Aβ levels by activating the PKA/SIRT1 signaling pathway. All the data are reported as mean ± *SEM* of four independent experiments. *p < 0.05 and **p < 0.01 versus the control group; #*p* < 0.05 and ##*p* < 0.01 versus the indicated group

cAMP, a molecule derived from ATP, is the first step in the activation of PKA, which can then phosphorylate a variety of targets, further controlling the biosynthesis of DNA and RNA (Hu et al., 2007). We found that PBMT significantly increased the levels of cAMP and ATP in SH‐SY5Y‐APPswe cells (Figure 6a,b). To elucidate whether an increase in cAMP level might be due to the increase in cellular ATP synthesis, cells were pretreated with ATP before 0 and 2 J/cm^2^ PBMT treatment. Our results showed that pretreatment with ATP followed by 2 J/cm^2^ PBMT treatment increased the cAMP level more than either PBMT treatment alone or treatment with exogenous ATP alone (Figure 6c). Furthermore, SH‐SY5Y‐APPswe cells were pretreated with oligomycin, an inhibitor of the electron transport chain complex V (ATP synthase), to inhibit ATP synthesis (Figure S7a). We found that oligomycin inhibited the increase in cAMP levels induced by PBMT, suggesting that ATP is necessary for PBMT‐induced increase in cAMP levels (Figure S7b).

The majority of cell chromophores including flavins, iron–sulfur centers, or heme are mainly concentrated in the mitochondrial complex enzymes, all of which have absorbance in the red to near‐infrared spectral range (Passarella & Karu, 2014). We first detected the activities of complexes I, II, III, IV, and V, which played a key role in oxidative phosphorylation and ATP production. As shown in Figure 6d, PBMT induced a dose‐dependent increase in complex IV (CcO) activity in SH‐SY5Y‐APPswe cells. Interestingly, no significant changes in complexes I, II, III, and V were observed (Figure S8a‐d). CcO is the enzyme that catalyzes the final step in the mitochondrial respiratory chain, and it has been reported as a mitochondrial photoacceptor upon which PBMT exerts its effect (Karu et al., 2005; Pastore et al., 2000). The mitochondrial membrane potential (ΔΨmt) is an important parameter, not only for mitochondria, but also for cellular status (Hu et al., 2007). We found that PBMT significantly enhanced ΔΨmt in a dose‐dependent manner in SH‐SY5Y‐APPswe cells by Rhodamine 123 labeling (Figure 6e,f). Meanwhile, the effects were blocked after treatment with sodium azide (NaN_3_; 10 mM), an inhibitor of CcO (Figure 6g,h). To further verify that CcO is responsible for the PBMT‐induced effects, SH‐SY5Y‐APPswe cells were transfected with siRNA targeting COX IV (Figure S8e), an essential nuclear coding subunit for assembly and respiratory function of the CcO (Li, Park, Deng, & Bai, 2006). We found that both pharmacological inhibition and gene knockout of CcO prevented not only the increases in ATP and cAMP (Figure 6i,j; Figure S8f,g), but also the activations of PKA and SIRT1 (Figure 6k; Figure S8h) in PBMT‐treated SH‐SY5Y‐APPswe cells.

## 3 DISCUSSION

To the best of our knowledge, this is the first report describing how near‐infrared PBMT of 632.8 nm is involved in protecting against spatial learning and memory impairments, diminishing senile plaque and Aβ levels by shifting APP processing toward the nonamyloidogenic pathway. The beneficial effects of PBMT are correlated with its ability to modulate CcO and activate the cAMP/PKA signaling pathway, leading to an enhancement of the deacetylase activity of SIRT1 (Figure 6l). Understanding the mechanism and functional significance of PBMT may lead to a potently promising therapeutic strategy for AD.

By far, the most attractive approach to AD treatment involves the development of drugs that in one way or another affect the stability, removal, or aggregation of Aβ, and clinically available pharmacotherapies of AD include acetylcholinesterase (AChE) inhibitors and N‐methyl‐D‐aspartate (NMDA) receptor antagonists (Bachurin, Bovina, & Ustyugov, 2017). Given that the etiology of AD is multifaceted, these agents only ameliorate certain symptoms, but do not change or delay the progression of AD, which substantially limits their clinical utility (Mangialasche et al., 2010). Thus, there is a dire need for new AD therapies. PBMT is nonthermal irradiation using light in the visible to near‐infrared range, which has been used clinically to reduce pain and inflammation in a variety of pathologies (Khuman et al., 2012). Our previous studies have shown that PBMT significantly diminished neuronal death induced by Aβ (Liang et al., 2012; Zhang et al., 2012). Moreover, PBMT could reverse dendritic atrophy via an increase in length and arborization in APP/PS1 neurons (Meng et al., 2013). This raised our interest to explore the potential protective role of PBMT in Aβ overproduction in AD.

As expected, we found that PBMT leads to a dramatic reduction in Aβ proportion in the cerebral cortex and hippocampus of APP/PS1 mice. Importantly, spatial learning and memory impairments were reversed by PBMT in APP/PS1 mice. Therefore, PBMT may have high clinical relevance. What caused the decrease in Aβ levels after PBMT treatment? Abnormally elevated Aβ levels in the brain are determined by its overproduction and decreased catabolism (Hardy & Selkoe, 2002). It has been documented that deficient degradation and clearance of Aβ contribute to its elevation in AD, which involves Aβ‐degrading enzymes, including endogenous NEP and IDE (Miners et al., 2008). Decreased IDE was observed in the APP/PS1 mice, but there were no significant changes in the protein levels of Aβ‐degrading enzymes after PBMT treatment. In addition, APP processing enzymes directly participate in Aβ production. We verified that PBMT up‐regulated ADAM10 and down‐regulated BACE1, which promoted the nonamyloidogenic pathway of APP in vivo and in vitro.

Recent studies have revealed that SIRT1 plays a pivotal role in mediating neuronal protection in animal models and cultured cells (Donmez, 2012; Herskovits & Guarente, 2014). Activation of SIRT1 enhances, whereas its loss‐of‐function impairs synaptic plasticity and memory formation (Gao et al., 2010). Loss of SIRT1 expression has been observed in AD, resulting in elevated Aβ production, whereas SIRT1 overexpression has been shown to decrease Aβ production, indicating that SIRT1 may have a profound effect on Aβ production (Shah et al., 2017). For instance, SIRT1 has been shown to up‐regulate ADAM10 expression by increasing the deacetylation level of RARβ, thereby promoting the nonamyloidogenic pathway of APP and attenuating Aβ generation in embryonic Tg2576 mouse neurons in vitro (Lee et al., 2014; Qin et al., 2006). In addition, SIRT1 could also activate PGC‐1α and further reduce Aβ production by down‐regulating BACE1 expression (Rodgers, Lerin, Gerhart‐Hines, & Puigserver, 2008; Wang et al., 2013). These data lead to a new hypothesis that the effects of PBMT on ADAM10 and BACE1 may be attributed to SIRT1‐mediated deacetylation. Our results support this hypothesis, since both pharmacological inhibition and knockdown of SIRT1 blocked PBMT‐mediated changes in ADAM10 and BACE1 expressions, further reversing the reduction of Aβ levels.

The cAMP/PKA pathway is involved in the regulation of SIRT1 activation and then participates in the regulation of Aβ production in vitro (Lee et al., 2014). It has been reported that the cAMP/PKA pathway rapidly activated SIRT1 to promote fatty acid oxidation (Gerhart‐Hines et al., 2011). Consistent with these studies, we found that PBMT enhances SIRT1 deacetylase activity by activating PKA in vitro. Furthermore, PBMT‐induced nuclear translocation of SIRT1 was also mediated by PKA.

How could PBMT activate the cAMP/PKA signaling pathway? PBMT could cause cAMP elevation, which depended on the energy available, as well as the mechanisms involved in the absorption of energy in the transduction of radiation into chemical energy (Hu et al., 2007). The most available energy in cells is the energetic ATP system. As a result, cAMP is synthesized from ATP by adenylate cyclase, and cAMP levels might be dependent on cellular ATP synthesis and concentration (Hu et al., 2007; Zhang, Zheng, Bradley, & Hexum, 2001). It has been suggested that PBMT‐induced biologic effects are caused by absorption of photons by intracellular photoacceptors, such as cytochrome c oxidase, which leads to electronically excited states, consequently resulting in acceleration of electron transfer reactions (Pastore et al., 2000). More electron transport necessarily leads to increased production of ATP, which further increases the level of cAMP to activate PKA (Hu et al., 2007; Mochizuki‐Oda et al., 2002). We confirmed that PBMT could enhance mitochondrial membrane potential and increase the levels of ATP and cAMP, via CcO activity. Importantly, PBMT‐induced activations of PKA and SIRT1 were also CcO dependent. It is worth noting that targeting CcO is an important pathway for improving AD, as mitochondrial dysfunction and decreased CcO activity have been observed in brains with AD (Perez‐Gracia, Torrejon‐Escribano, & Ferrer, 2008).

Overall, the current investigation demonstrates that PBMT can effectively penetrate into the brain, thereby reversing spatial learning/memory impairments and reducing senile plaque/Aβ levels in AD model mice, which has great potential for transformation. Importantly, mechanistic studies reveal that the dramatic Aβ reduction caused by PBMT is attributed to its ability to shift APP processing toward the nonamyloidogenic pathway through activation of the PKA/SIRT1 pathway. A better understanding of the regulation mechanism of photobiomodulation may provide a therapeutic strategy to control the progression of AD.

## 4 EXPERIMENTAL PROCEDURES

### 4.1 Animals

The double transgenic mice (APPswe/PSENdE9) were purchased from The Jackson Laboratory. The detailed descriptions about animals reference the Supporting Information.

### 4.2 Cultures of primary neurons and human SH‐SY5Y neuroblastoma cells

Cultures of primary hippocampal neurons and SH‐SY5Y cells were performed as previously described (Meng et al., 2013). See the Supporting Information for details.

### 4.3 Reagents and antibodies

For all chemical reagents used in this study, see the Supporting Information. Primary antibodies, their sources, and the concentrations used are listed in Table S1.

### 4.4 PBMT treatment in vivo and in vitro

All of the six‐month‐old mice of PBMT group were placed in the mouse fixator following head hair removal (without removing the scalp and skull) and only exposed their head and tail, while the optical fiber is located above the head of mice, and the tail does not receive He‐Ne laser (HN‐1000; Laser Technology Application Research Institute, Guangzhou, China). We measured that the transmittance of 632.8 nm PBMT penetrating the scalp and skull to the interior of the hippocampus was approximately 30%. Mice received the continuous laser for 10 min at an irradiation power of 92 mW, with the corresponding fluences of 2 J/cm^2^ reaching the interior of the hippocampus by measurement (without local temperature increase in the scalp and brain tissue). The control groups were maintained in the same mouse fixator for the same amount of time as the irradiated groups, but the laser source was not activated (sham irradiation). The most important laser parameters were reported in vivo study according to a previous study (Jenkins & Carroll, 2011): wavelength = 632.8 nm; power = 92 mW; irradiation time = 10 min; beam area at the skin = 0.785 cm^2^; pulse parameters = continuous; anatomical location = cerebral cortex and hippocampus; number of treatments = 30; and frequency of treatments = once a day. PBMT treatment with cells was conducted as described in our previous study (Liang et al., 2012). Briefly, cells were irradiated with He‐Ne laser for 0.7, 1.25, 2.5, and 5 min at an irradiation power of 10 mW in the dark, with the corresponding fluences of 0.5, 1, 2, and 4 J/cm^2^, respectively. The calculation formula of designated time for PBMT treatment is time (s) = energy (J/cm^2^) × surface (cm^2^)/power (W) (Meng et al., 2013). Throughout each experiment, cells were kept either in a complete darkness or in an extremely dim environment, except when subjected to the light irradiation, to minimize ambient light interference. For the measurement of PBMT transmittance in animal experiments as described previously (Henderson & Morries, 2015), see Supporting Information and Figure S9 for details. For complete parameters including device information, irradiation parameters and treatment parameters used in PBMT treatment in vivo and in vitro, see Tables S2–S4.

### 4.5 Morris Water Maze (MWM) Test

After PBMT treatment, hippocampus‐dependent spatial learning and memory abilities were evaluated with the MWM as previously described (Trinchese et al., 2004), with some modifications. See the Supporting Information for details.

### 4.6 Quantification of Aβ levels

Quantitative assessment of Aβ peptides in the cortex and hippocampus of APP/PS1 mice or Aβ peptides secretion in neurons was performed according to the protocol of the human Aβ_1‐40_ and Aβ_1–42_ ELISA kits (Invitrogen, USA) as previously described (Qin et al., 2006). See the Supporting Information for details.

### 4.7 SIRT1 deacetylase activity assays

SIRT1 deacetylase activity was measured using the fluorometric SIRT1 assay kit (Sigma‐Aldrich, CS1040) in accordance with the instructions of manufacture. Details of SIRT1 activity assays were displayed in the Supporting Information.

### 4.8 Western blotting and co‐immunoprecipitation (co‐IP)

Western blotting and co‐IP were performed following our previous description with some modifications (Zhang, Liu, Wu, & Xing, 2016). See the Supporting Information for details.

### 4.9 Immunohistochemistry and Immunocytochemistry

Immunohistochemistry and immunocytochemistry were performed as described previously (Meng et al., 2013). See the Supporting Information for details.

### 4.10 RNAi‐mediated gene silencing and Semi‐quantitative RT‐PCR analysis

RNAi‐mediated gene silencing and semi‐quantitative RT‐PCR experiments were performed as previously described (Meng et al., 2013). See the Supporting Information for details.

### 4.11 ELISA assays for ATP and cAMP detection

ELISAs were performed to measure the levels of cellular ATP and cAMP. See the Supporting Information for details.

### 4.12 Complexes I, II, III, IV, and V activity assays

Mitochondrial electron transport chain complexes I, II, III, IV, and V activities were assayed using the commercial activity detection kits in accordance with the instructions of manufacture. Details were displayed in the Supporting Information.

### 4.13 Evaluation of mitochondrial membrane potential (ΔΨmt)

The mitochondrial membrane potential was determined using the fluorescent cationic dye, Rhodamine 123. See the Supporting Information for details.

### 4.14 Experimental design and statistical analysis

Quantified data presented in all figures are from one representative experiment among at least three independent experiments and are expressed as the mean ± *SEM*. Statistical details for each experiment are included in the figure legends. Significant differences between groups were compared using the one‐way ANOVA procedure, followed by a Student *t* tests using SPSS software (IBM). Differences were considered statistically significant at *p* < .05.

## CONFLICT OF INTEREST

None declared.

## AUTHOR CONTRIBUTIONS

Z.Z., Q.S., and D.X. designed the research; Z.Z., Q.S., and X.W. performed the experiments; Z.Z., Q.S., D.Z., and D.X. analyzed the data; and Z.Z., Q.S., and D.X. wrote the article.
