# Supplementary material for: Activation of PKA/SIRT1 signaling pathway by photobiomodulation therapy reduces Aβ levels in Alzheimer's disease models
Source: Aging Cell. 2019 Oct 30;19(1):e13054. doi: 10.1111/acel.13054 (PMC6974721; doi:10.1111/acel.13054)
Supplement: Supplementary file 1 [file ACEL-19-e13054-s001.doc]

**Supporting Information**

**Supplementary Figures**

**
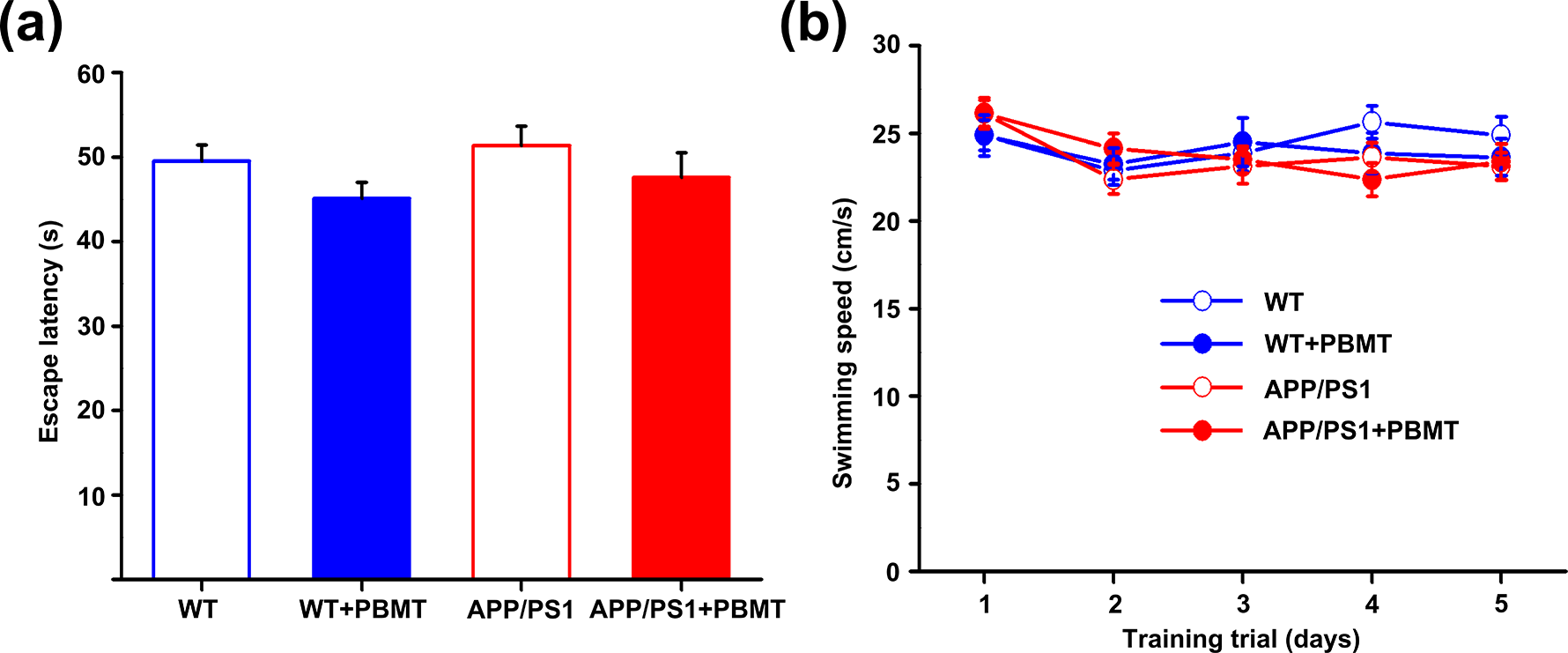
**

**Figure S1.** Effects of PBMT on the escape latency in the visual platform trial and the swimming speed in the hidden platform task. (a) Visible platform task on the first day showing no difference was observed for escape latency among the four groups of mice. (b) The average swimming speed of mice to find the hidden platform was recorded on each training day. All the data in these figures are reported as mean ± SEM. n = 9 animals per group.


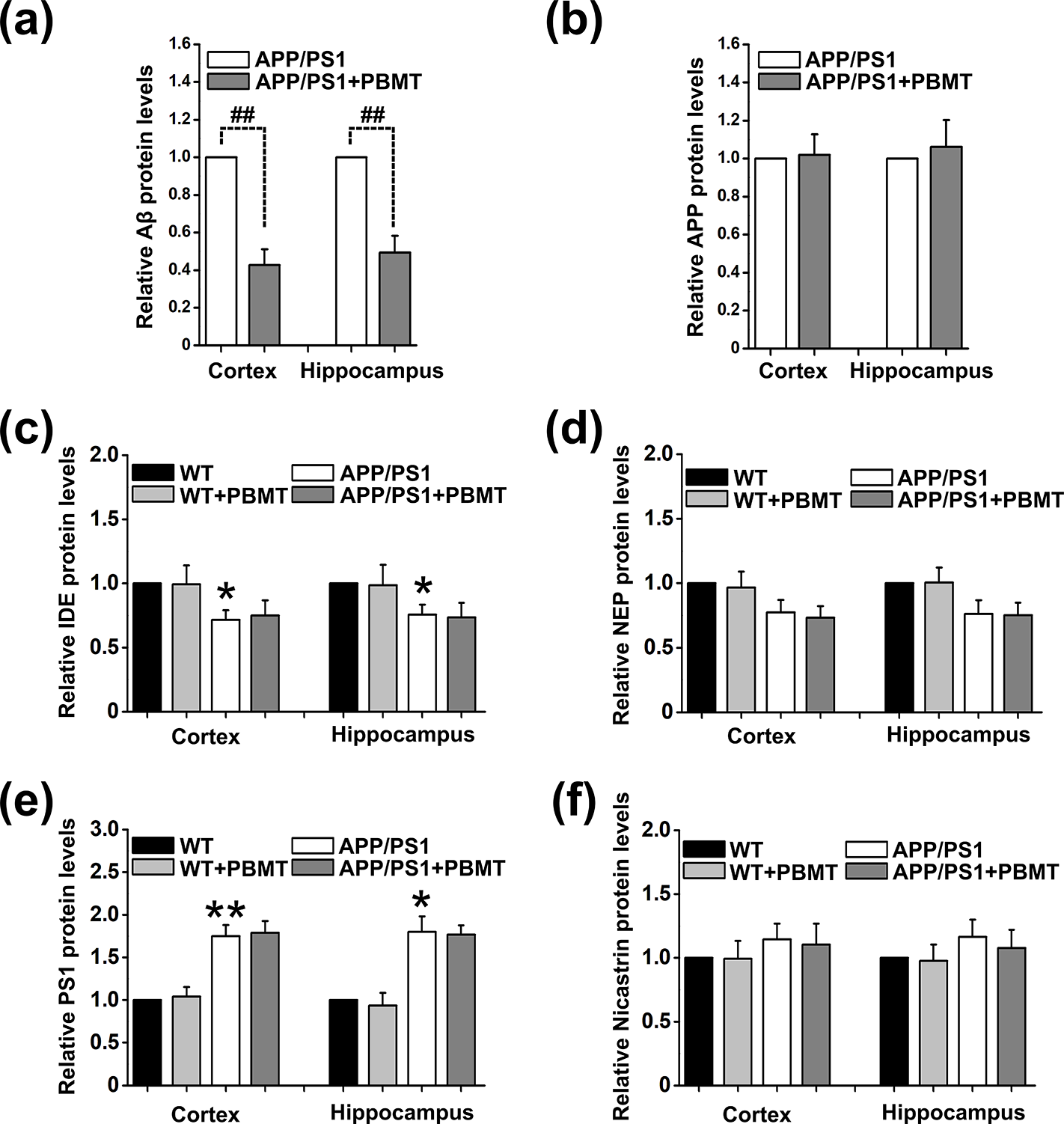


**Figure S2.** Effects of PBMT on APP processing and Aβ degradation in APP/PS1 mice. (a–f) Densitometric quantification of exogenous human Aβ (a) and APP (b), endogenous mouse IDE (c), NEP (d) and nicastrin (f), and both endogenous and exogenous PS1 (e) expressions after indicated treatments in APP/PS1 mice. Data are reported as mean ± SEM. n = 5 animals per group. **p* < 0.05 and ***p* < 0.01 versus the control group; ##*p* < 0.01 versus the indicated group.


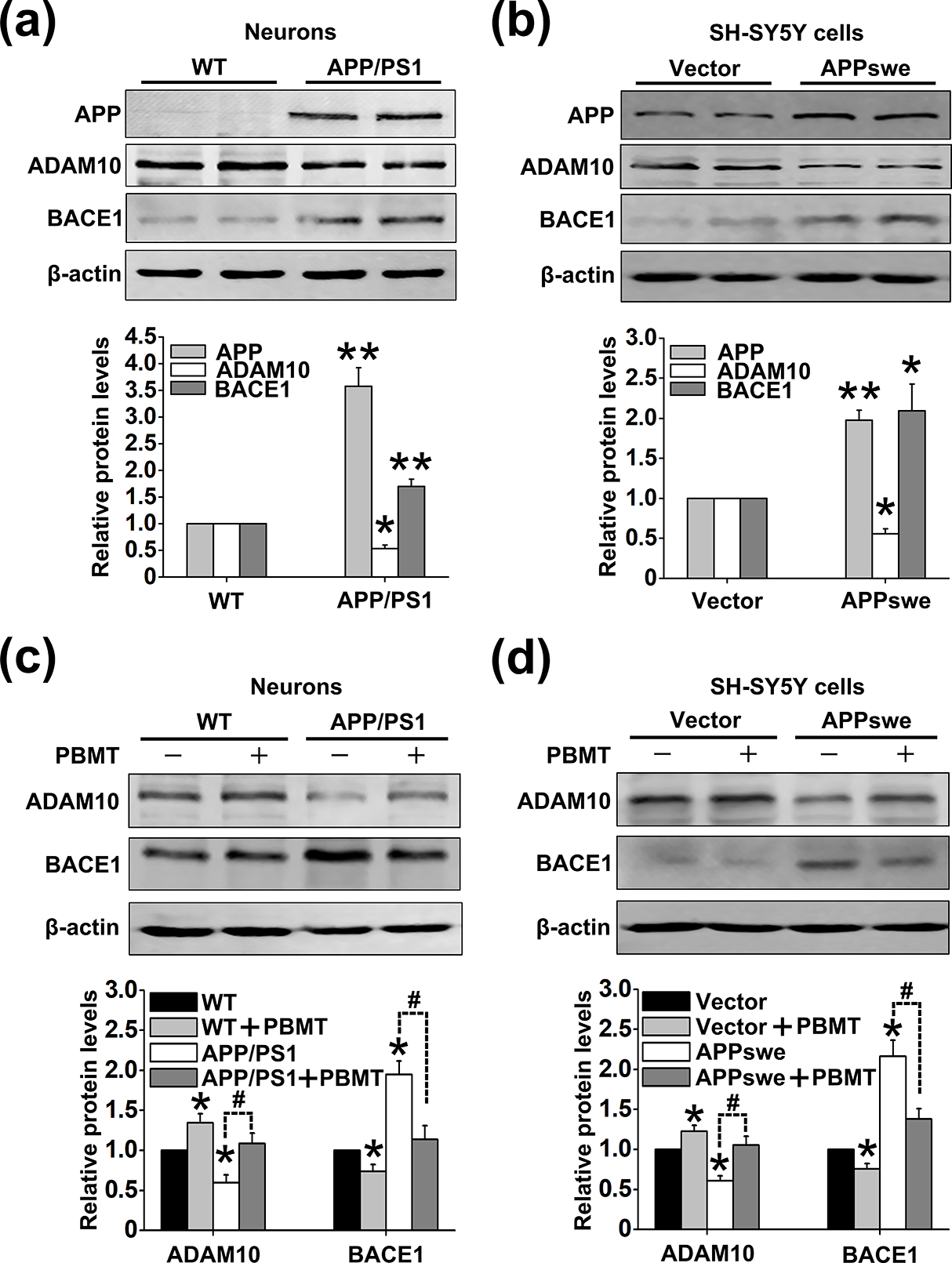


**Figure S3.** PBMT increases ADAM10 protein levels and decreases BACE1 protein levels in APP/PS1 neurons and SH-SY5Y-APPswe cells. (a) Western blot analysis of exogenous APP, endogenous ADAM10, and endogenous BACE1 levels in primary hippocampal neurons derived from WT and APP/PS1 mice. (b) Western blot analysis of both endogenous and exogenous APP, endogenous ADAM10, and endogenous BACE1 levels in SH-SY5Y and SH-SY5Y-APPswe cells. (c, d) Western blotting was performed to detect the ADAM10 and BACE1 expressions with or without PBMT (2 J/cm^2^) in primary neurons (c) and SH-SY5Y cells (d). All the data are reported as mean ± SEM of four independent experiments. **p* < 0.05 and ***p* < 0.01 versus the control group; #*p* < 0.05 versus the indicated group.


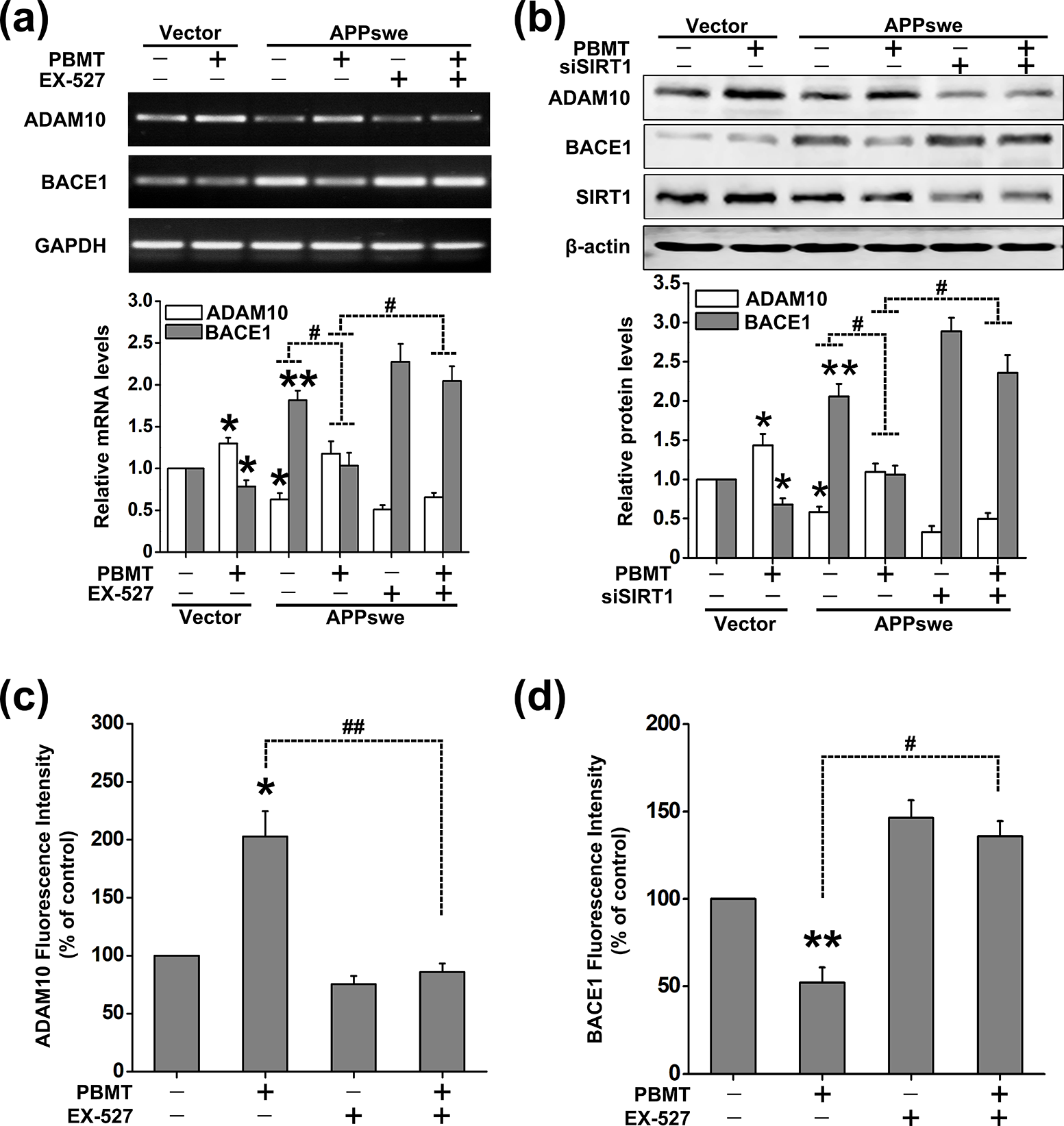


**Figure S4.** PBMT up-regulates ADAM10 and down-regulates BACE1 via activation of SIRT1 in APP/PS1 neurons and SH-SY5Y-APPswe cells. (a) RT-PCR analysis of ADAM10 and BACE1 mRNA levels after PBMT treatment in the presence of EX-527 (20 μM) in SH-SY5Y-APPswe cells. (b) Western blot analysis of ADAM10 and BACE1 expressions stimulated with PBMT after transfection with SIRT1 siRNA in SH-SY5Y-APPswe cells. (c, d) The fluorescence intensity data of ADAM10 (c) and BACE1 (d) were recorded by confocal microscopy. All the data in these figures are reported as mean ± SEM of four independent experiments. **p* < 0.05 and ***p* < 0.01 versus the control group; #*p* < 0.05 and ##*p* < 0.01 versus the indicated group.


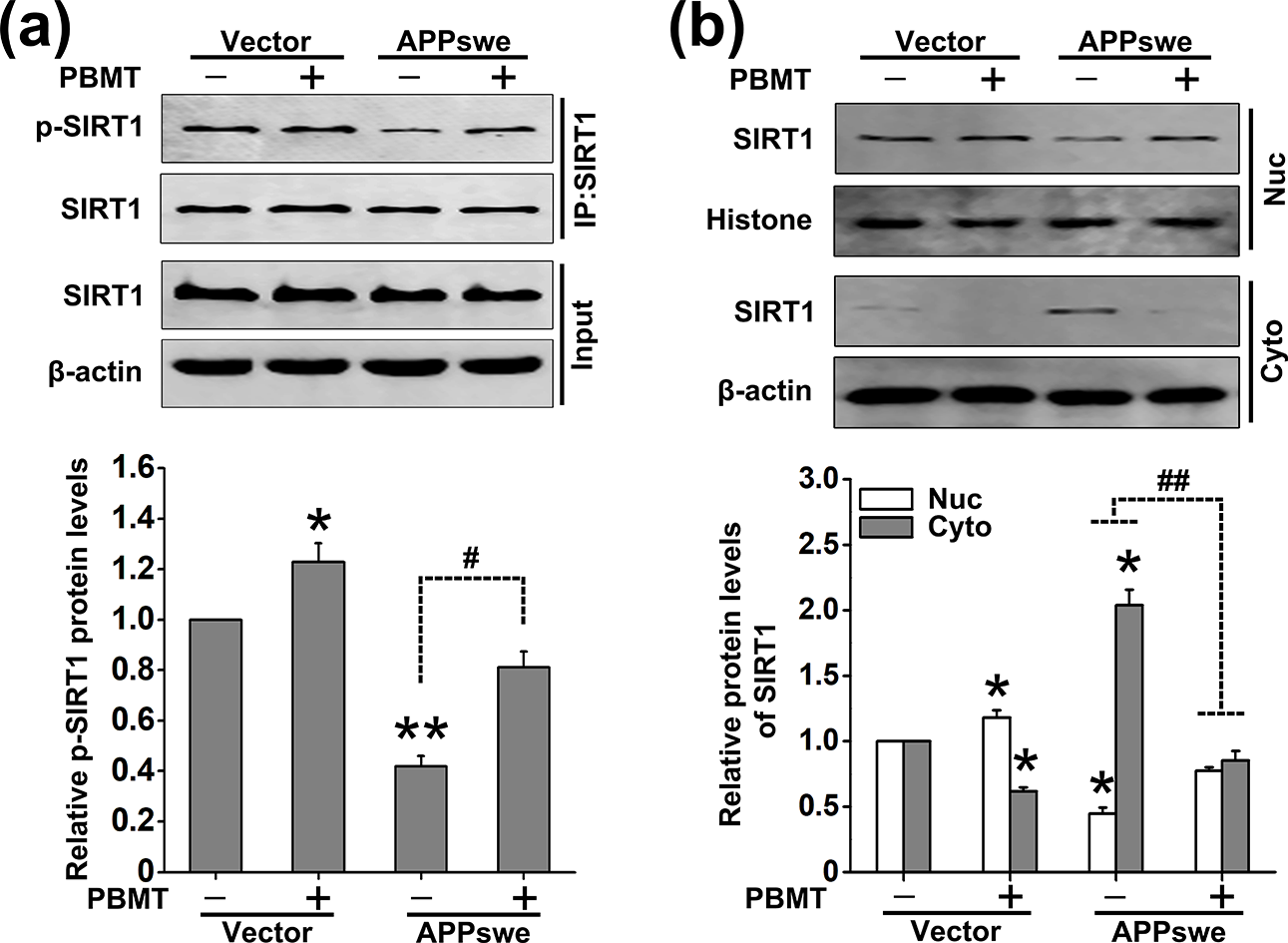


**Figure S5.** PBMT activates SIRT1 and promotes its translocation to the nucleus. (a) Immunoprecipitates were analyzed to detect SIRT1 serine phosphorylation levels by western blotting with anti-SIRT1 and anti-p-Ser antibodies in SH-SY5Y and SH-SY5Y-APPswe cells after PBMT treatment. (b) Representative western blot assay for detecting the levels of SIRT1 after indicated treatments in cytoplasm and nuclear lysates of SH-SY5Y-APPswe cells, respectively. All the data are reported as mean ± SEM of four independent experiments. **p* < 0.05 and ***p* < 0.01 versus the control group; #*p* < 0.05 and ##*p* < 0.01 versus the indicated group.


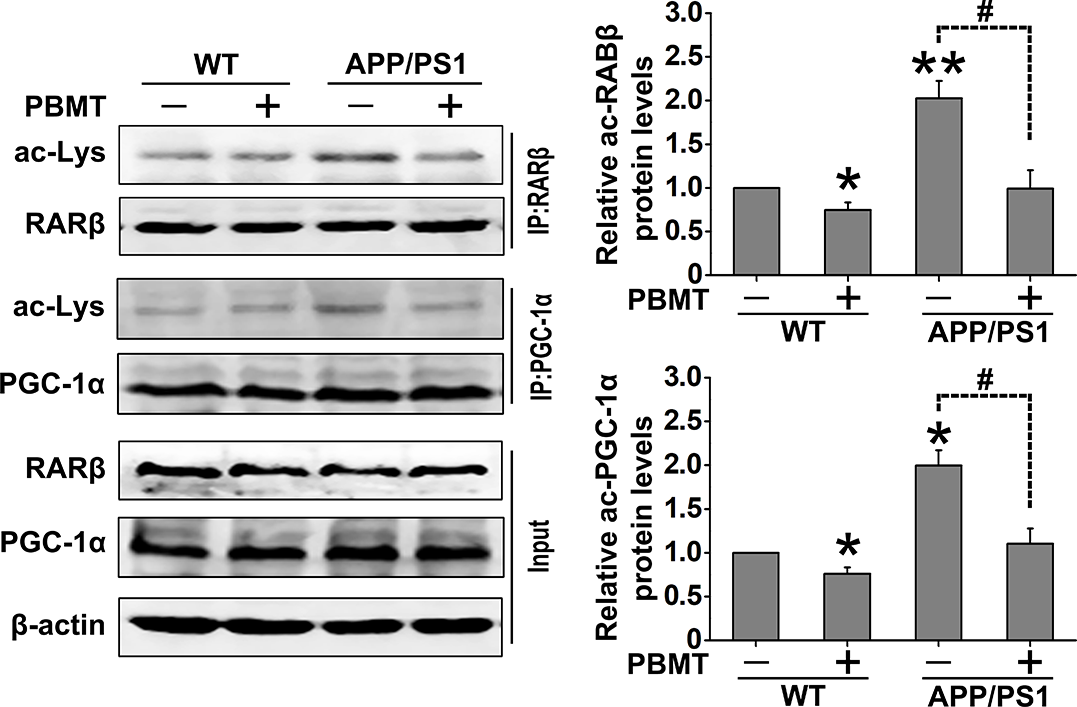


**Figure S6.** PBMT reduces the acetylation levels of RARβ and PGC-1α *in vivo*. Immunoprecipitates were analyzed to detect the tyrosine acetylation levels of RARβ and PGC-1α by western blotting with RARβ, PGC-1α, and Ac-K (anti-pan acetylated lysine) antibodies in PBMT-treated APP/PS1 transgenic mice and age matched WT mice at 6 months of age. All the data are reported as mean ± SEM. n = 5 animals per group. **p* < 0.05 and ***p* < 0.01 versus the control group; #*p* < 0.05 versus the indicated group.


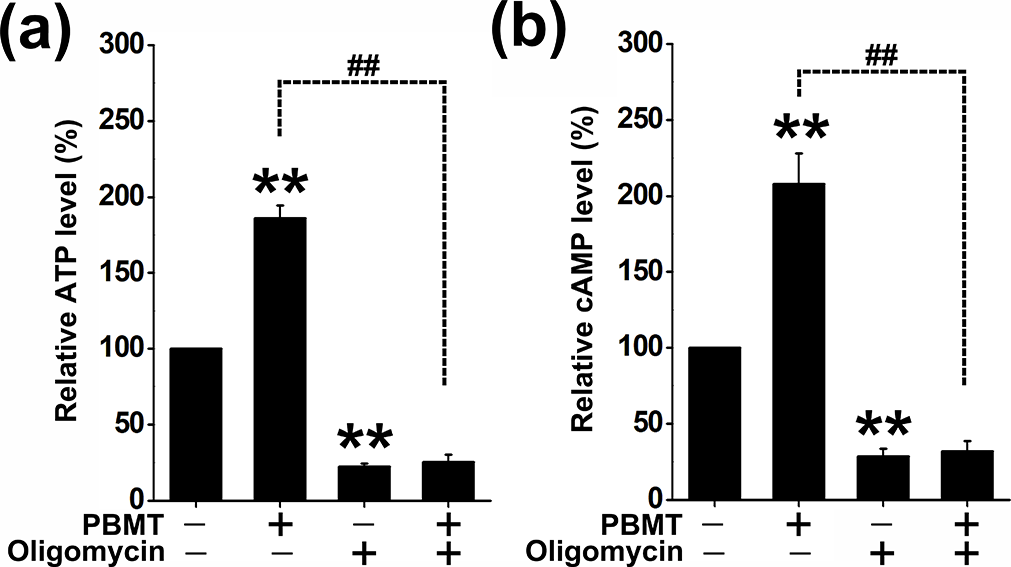


**Figure S7.** Inhibiting ATP synthesis blocks the PBMT-induced increase in cAMP levels. (a, b) SH-SY5Y-APPswe cells were treated with oligomycin (20 μM) for 30 min. ELISAs were performed to detect ATP and cAMP levels after treatment with PBMT in the presence of oligomycin in SH-SY5Y-APPswe cells. Relative ATP (a) and cAMP (b) levels were calculated as the percentage of the control group level. All the data are reported as mean ± SEM of four independent experiments. ***p* < 0.01 versus the control group; ##*p* < 0.01 versus the indicated group.


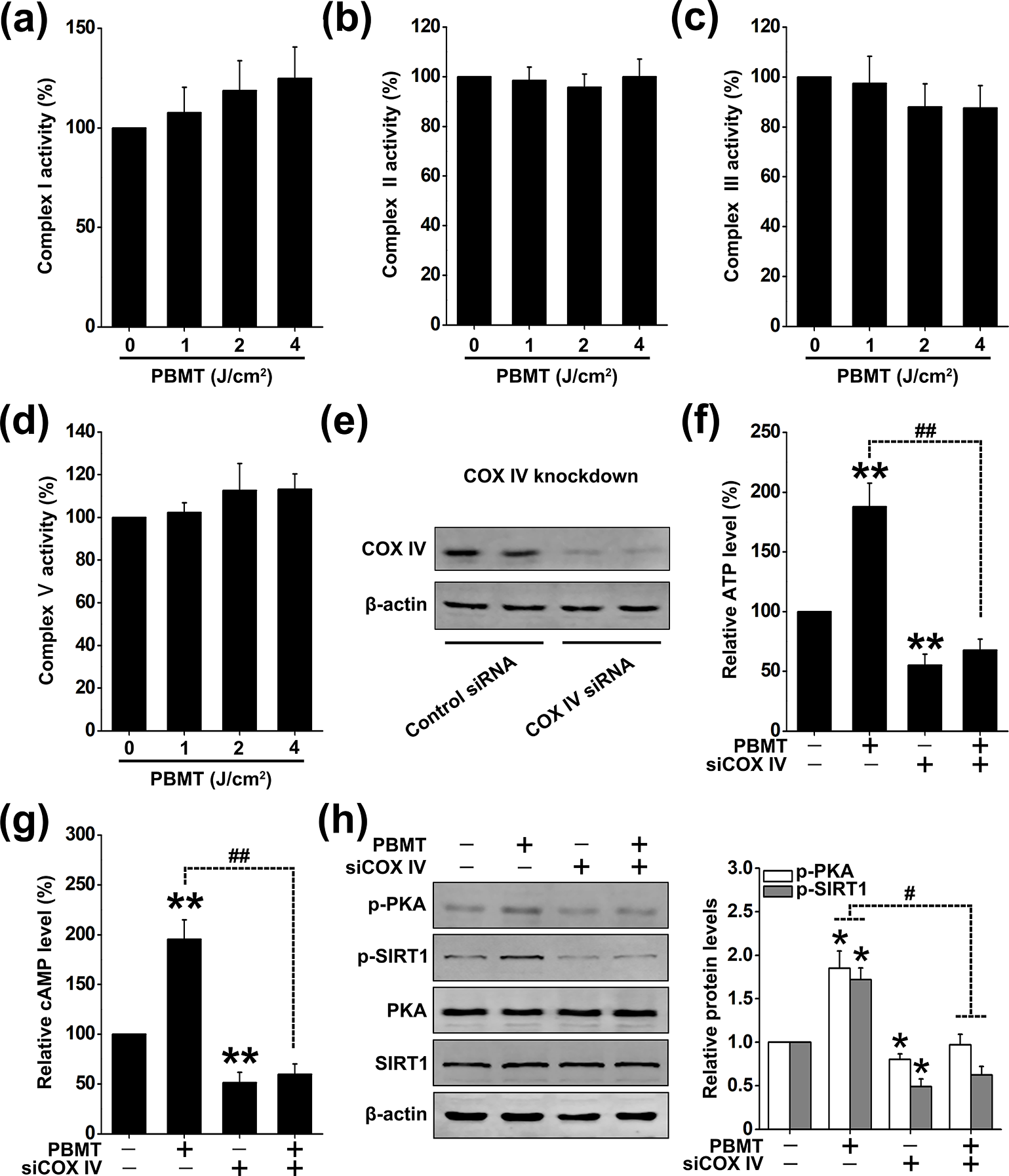


**Figure S8.** Mitochondrial photoacceptor CcO activity is necessary for PBMT-induced increases in ATP and cAMP levels and activations of PKA and SIRT1. (a-d) SH-SY5Y-APPswe cells were treated with 0, 1, 2, and 4 J/cm^2^ PBMT. Relative electron transport chain complexes I, II, III, and V activities were detected. (e) COX IV knockdown in SH-SY5Y-APPswe cells. (f, g) SH-SY5Y-APPswe cells were transfected with COX IV siRNA. ELISA assays were performed to detect ATP and cAMP levels. Relative ATP (f) and cAMP (g) levels were calculated as the percentage of the control group level. (h) Western blot analysis of p-PKA and p-SIRT1 expressions after COX IV siRNA transfection in PBMT-treated SH-SY5Y-APPswe cells. All the data are reported as mean ± SEM of four independent experiments. **p* < 0.05 and ***p* < 0.01 versus the control group; #*p* < 0.05 and ##*p* < 0.01 versus the indicated group.


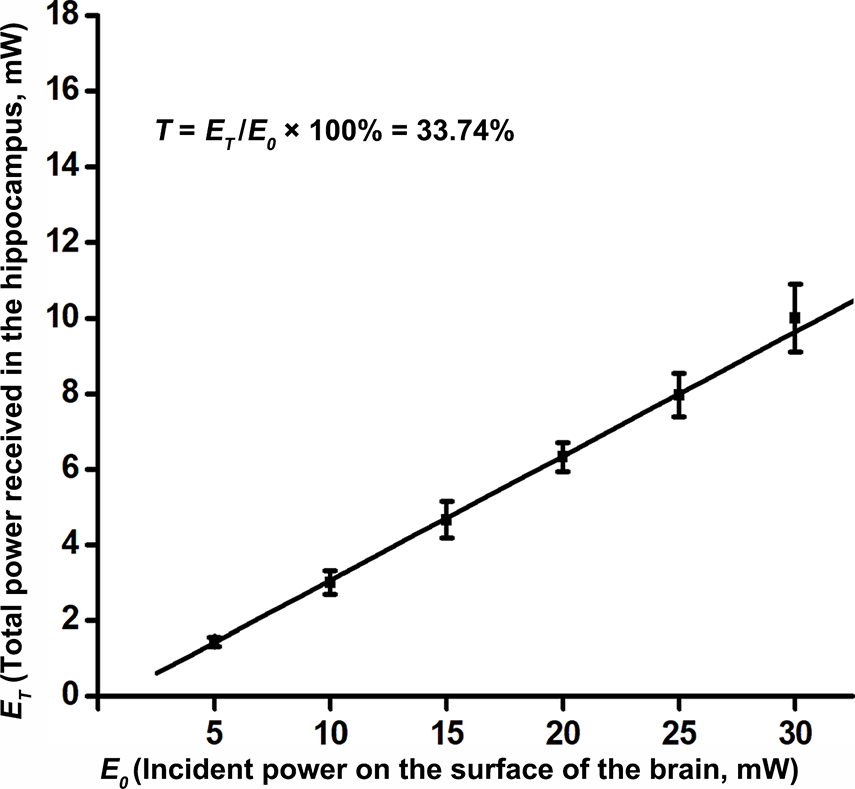


**Figure S9.** PBMT transmittance is measured from the upper part of the exposed brain to the interior of the hippocampus. The brain of six-month-old mice (n = 5) was separated and transected to expose the hippocampus. The upper part of the brain was irradiated with laser of different energies. We measured the energy that penetrated the cortex to reach the hippocampus by using a power meter. The following equation was used to calculate transmittance: *T* = *E_T_*/*E_0_* × 100%, in which *T* is the transmittance, *E_T_* is the total energy received in the hippocampus transmitting through the brain. *E_0_* is the incident energy on the surface of the brain.

**Supplementary Tables**

**Table S1.** List of antibodies used in this study.

| Target antibody | Source | Catalog no. | Application/dilution |
| --- | --- | --- | --- |
| APP  Aβ  sAPPα  sAPPβ  ADAM10  BACE1  PS1  Nicastrin  NEP  IDE  SIRT1  PGC-1α  RARβ  PKAα/β/γ  p-PKAα/β/γ (T197)  acetyl Lysine  Phosphoserine  Histone  COX IV  β-actin | Cell Signaling Technology  Covance  Immuno-Biological Laboratories  Immuno-Biological Laboratories  Abcam  Abcam  Cell Signaling Technology  Cell Signaling Technology  Millipore  Abcam  Cell Signaling Technology  Abcam  Thermo Fisher-Invitrogen  Santa Cruz Biotechnology  Abcam  Abcam  Millipore  Cell Signaling Technology  Cell Signaling Technology  Santa Cruz Biotechnology | 2450  SIG-39320  11088  10321  ab1997  ab108394  5643  30239  AB5458  ab133561  8469  ab191838  MA1-811  sc-365615  ab75991  ab22550  05-1000  4499  4850  sc-47778 | WB/1:500  ICC/1:200  WB/1:1000  IHC/1:300  WB/1:500  WB/1:500  WB/1:1000  ICC/1:500  WB/1:1000  ICC/1:500  WB/1:1000  WB/1:1000  WB/1:1000  WB/1:1000  WB/1:1500  ICC/1:500  co-IP/1:200  WB/1:1000  co-IP/1:200  WB/1:1000  co-IP/1:200  WB/1:500  WB/1:2000  WB/1:1000  WB/1:1000  WB/1:1000  WB/1:1000  WB/1:1000 |

WB, Western blot; ICC, Immunocytochemistry; IHC, Immunohistochemistry.

**Table S2.** Device information used in PBMT treatment.

| Manufacturer  Model Identifier  Number of Emitters  Emitter Type  Beam Delivery System | Laser Technology Application Research Institute  HN-1000  1  He-Ne Laser  Fiberoptic |
| --- | --- |

**Table S3.** Specifications for irradiation parameters.

| Parameter (unit) | Value |
| --- | --- |
| Center wavelength (nm)  Spectral bandwidth (nm)  Operating mode  Frequency (Hz)  Average radiant power (mW)  Polarization  Beam divergence  Beam shape  Beam profile | 632.8 nm  No (Single wavelength, no bandwidth)  Continuous wave  No (Continuous wave, no frequency)  92 mW for mice and 10 mW for cells  No  < 2 rad for a Gaussian source  Circular  Top Hat (We designed an engineering diffuser which efficiently converted a collimated Gaussian beam to a Top Hat beam) |

**Table S4.** Specifications for treatment parameters.

| Parameter (unit) | Value for mice | Value for cells |
| --- | --- | --- |
| Beam spot size at target (cm^2^)  Exposure duration (sec)  Radiant exposure (J/cm^2^)  Radiant energy (J)  Number of points irradiated  Application technique  Number of treatment sessions  Frequency of treatment sessions  Total radiant energy (J) | 0.785 cm^2^  600 sec  2 J/cm^2^  1.57 J  1  Without skin contact  30 sessions  Once a day  1 J per session, 47.1 J over all sessions | 9.6 cm^2^  150 sec  2 J/cm^2^  19.2 J  1  Without surface contact  1 session |

**Supplementary Experimental Procedures**

**Animals**

The characterization of the APP/PS1 double transgenic mice, expressing a chimeric mouse/human amyloid precursor protein bearing the Swedish mutation (Mo/HuAPP695swe) and a mutant human Presenilin 1 protein (PS1-dE9) in central nervous system neurons, has been described previously . The genotype was confirmed by polymerase chain reaction (PCR) analysis of tail biopsies. To more closely mimic the clinical setting, in the present study, we used six-month-old male APP/PS1 mice with preexisting a subset of behavioral and pathological features of AD and their non-transgenic wild-type (WT) littermates as control. All of the experimental mice were of the C57BL/6 background, and the WT and transgenic mice were paired from the litters and housed under the same living conditions.

The present study was performed following the Guide for the Care and Use of Laboratory Animals (Institute of Laboratory Animal Resources, Commission on Life Sciences, National Research Council). This study was approved by the Institutional Animal Care and Use Committee of our university (South China Normal University, Guangzhou, China).

**Primary neuronal culture**

Primary neuronal culture derived from hippocampal of mice embryonic day 14 was performed as previously described . Neurons were seeded in polylysine-coated wells and maintained in neurobasal medium supplemented with 2% B27 (Invitrogen, USA) + 2 mM L-glutamine, penicillin (100 U/mL), and streptomycin (100 μg/mL). For the identification of APP/PS1 transgenic neurons, the genotype of the animals was determined by PCR using DNA obtained from fibroblasts. All of the cultures were kept at 37°C in a humidified 5% CO_2_ containing atmosphere.

**Human SH-SY5Y neuroblastoma cell culture**

Human SH-SY5Y neuroblastoma cell culture was performed as previously described . Cells were maintained in Dulbecco’s modified Eagle’s medium (Gibco, CA, USA), containing 10% fetal bovine serum (Gibco) and antibiotics (penicillin and streptomycin) in 5% CO_2_ at 37°C in a humidified incubator. SH-SY5Y cells were transfected with an empty pcDNA 3.1 vector containing a cytomegalovirus promoter, an APP Swedish KM670/671NL double mutation (APPswe), or control vector (APP695) using Lipofectamine 3000 (Thermo Fisher-Invitrogen, Carlsbad, CA, USA), according to the manufacturer’s instructions. To obtain stable cell lines, single-cell clones were generated by selection with 500 μg/mL G418 (Sigma-Aldrich, USA), and the transfection/expression of the target genes were identified by western blot analysis.

**Reagents and antibodies**

The following reagents were used: Resveratrol, EX-527 and oligomycin were purchased from MCE. PD98059 and API-2 were purchased from Santa Cruz Biotechnology. Gӧ6983 was purchased from Merck. Thioflavin T, 4′ 6-diamidino-2-phenylindole (DAPI), Actinomycin D, H-89, and sodium azide (NaN_3_) were obtained from Sigma-Aldrich.

Primary antibodies, their sources, and their respective dilutions are listed in Table S1. Alexa Fluor-conjugated antibodies used in western blotting and immunostaining were from Abcam.

**Measurement of PBMT transmittance in animal experiments**

Our previous studies have shown that laser with a dose of 2 J/cm^2^ is particularly effective in improving neuronal functions . In animal experiments, in order to be received the same dose of laser in the hippocampus, an important region for learning and memory that is vulnerable to AD, we previously measured and calculated the actual power of the laser after penetrating the skin and skull into the interior of the hippocampus. Penetration of PBMT through brain tissues is determined by energy and attenuation coefficient . First, we removed brain tissues from the head of the six-month mice and measured the transmittance of PBMT from the upper part of the exposed brain to the interior of the hippocampus. We found that 632.8 nm PBMT had an approximately 30% transmittance (Figure S9), which indicated that 6 J/cm^2^ PBMT irradiation is needed above the brain. We next measured the actual power of the laser irradiation on the head in animal experiments, making the dose of laser penetrating the epidermis and skull to the bottom of the skull 6 J/cm^2^, which was designed to allow the hippocampus to receive 2 J/cm^2^ PBMT. A penetrating dose of 2 J/cm^2^ reaching the interior of the hippocampus was used in animal experiments, which is consistent with the dose of PBMT used *in vitro*.

**Morris Water Maze (MWM) Test**

After PBMT treatment, hippocampus-dependent spatial learning and memory abilities were evaluated with the MWM as previously described , with some modifications. Briefly, the water maze was divided into four quadrants. A hidden platform was placed 2 cm below the water surface in the center of one quadrant during training. Mice were subjected to training trials (the navigation test), followed by probing without the platform. During the training trials, mice were released into the maze from a randomly selected quadrant, with all animals using the same order. Escape latency to find the underwater platform was recorded and each animal performed five training sessions from different starting quadrants per day. The escape latencies (seconds) from trials in each daily session were averaged for each mouse. For the probe trial on day 6, the platform was removed from the pool, and the animals were subjected to a 60 s probe trial following the last training session to find the original platform. The proportion of time spent in the target quadrant and platform location crossings were monitored and recorded by a video camera linked to a computer-based image analyzer.

**Quantification of Aβ levels**

Aβ_1–40_ and Aβ_1–42_ peptides in the cortex and hippocampus of APP/PS1 mice were measured according to the protocol of the human Aβ_1-40_ and Aβ_1–42_ ELISA kits (Invitrogen, USA). Briefly, frozen mouse brain homogenates were sequentially extracted initially in freshly-prepared Tris-buffered saline (TBS; 20 mM Tris-HCl, 150 mM NaCl, PH 7.4) containing protease and phosphatase inhibitors, and homogenized on a mechanical Fluka homogenizer, according to a previously published method . The homogenate was centrifuged at 20,000 g for 30 min at 4°C. The supernatant containing soluble Aβ peptides was collected and stored at -80°C. The sediment containing insoluble Aβ peptide was re-homogenized with an equal volume of TBS, plus 5 M guanidine HCl, and incubated at room temperature. After re-centrifugation, the supernatant was collected and stored at -80°C. Soluble and insoluble Aβ levels were determined employing the human Aβ_1-40_ and Aβ_1–42_ ELISA kits.

For quantitative assessment of Aβ peptides secretion in APP/PS1 neurons, conditioned media from neurons were centrifuged at 3,500*g* for 10 min at 4°C to remove cellular debris. Aβ_1-40_ and Aβ_1–42_ were assessed by ELISA as described above.

**SIRT1 deacetylase activity assays**

SIRT1 deacetylase activity was measured using the fluorometric SIRT1 assay kit (Sigma-Aldrich, CS1040), following the manufacturers recommendations. Briefly, cells were lysed in RIPA buffer containing Trichostatin A (TSA, 5 μM) to block histone deacetylase activity and then incubated for 10 min at 37°C to allow degradation of any NAD^+^ contaminants. The extracts (30 μg protein per reaction) were then incubated with 10 μL of the SIRT1 substrate solution in the presence or absence of NAD^+^. Plates were incubated at 37°C for 1 h, 5 μL of developing buffer were added to each well, and the plates were incubated at 37°C for 10 min. Fluorescence intensity (excitation wavelength: 360 nm, emission wavelength: 450 nm) was measured using a FLUOstar Omega Plate Reader (BMG Labtech, Ortenberg, Germany). The SIRT1 inhibitor nicotinamide was used to confirm the specificity of the reaction; the fluorescence values obtained in the absence of NAD^+^ did not differ from the blank. SIRT1-dependent deacetylase activity was calculated after subtracting fluorescence values obtained in the absence of NAD^+^.

**Western blotting and co-immunoprecipitation (co-IP)**

Western blotting was performed following our previous description with some modifications . Briefly, equivalent proteins were loaded on SDS-PAGE, transferred to a PVDF membrane (Roche Applied Sciences, Indianapolis, IN, USA), and blotted with indicated primary antibodies, followed by Alexa Fluor-conjugated secondary antibodies. Detection was performed using the Odyssey Infrared Imaging System (LI-COR, Biosciences, Lincoln, NE, USA). The intensity of the western blot signals was quantified using ImageJ software (National Institutes of Health, Bethesda, MD). The results of densitometry analyses are presented as the ratio of protein/β-actin protein, are compared with controls, and normalized to 1.

For co-IP, protein samples were incubated with the indicated antibody at 4°C for 2–4 h, and then incubated with 50% slurry of protein A-Sepharose (Roche Applied Sciences) at 4°C overnight. The beads were washed three times and collected by centrifugation at 12,000*g* for 5 s. After the final wash, the beads were re-suspended with SDS sample buffer, boiled for 5 min and analyzed by western blotting.

**Immunohistochemistry**

After MWM, all animals were deeply anesthetized with sodium pentobarbital (50 mg/kg intraperitoneally), and mice were perfused transcardially with ice-cold 0.01M PBS (pH 7.4). The left hemispheres of brains were snap frozen for protein extraction, and the right hemispheres were fixed in 4% paraformaldehyde (pH = 7.4) for 48 hours and dehydrated through the gradient of 15% and 30% sucrose solutions until sank at 4°C. The brains were mounted in Optimal Cutting Temperature (OCT) compound (AO, USA), and sequential coronal brain sections (10 μm thick) were obtained and mounted on Superfrost Plus glass slides (Thermo Fisher-Invitrogen). For immunostaining, the sections were permeabilized with 0.5% Triton X-100 in PBS for 30 min and blocked with 5% bovine serum albumin at 37°C for 1 h and subsequently incubated in appropriately diluted primary anti-Aβ antibody overnight at 4°C. After washing with TBS/0.025% Tween 20, the sections were incubated with diluted Alexa Fluor-conjugated secondary antibodies for 2 hours at room temperature. After washes, sections were mounted and coverslipped with DAPI. Thioflavin T staining for fibrillar Aβ plaques was performed by incubating slides in 0.5% Th-T, followed by rinsing in ethanol and distilled water. The green fluorescent protein-stained plaques were visualized using fluorescence microscopy. For quantification analyses, microscopic images were used to calculate the percentage of area occupied by Aβ-positive plaques and Th-T-positive plaques by using Image-Pro Plus imaging software (Media Cybernetics, Bethesda, MD, USA).

**Immunocytochemistry**

Immunocytochemical staining was performed as described in a previous study . After being treated under different experimental conditions, cells were fixed in 4% paraformaldehyde in PBS for 15 min at room temperature and were then membrane-permeabilized with 0.5% TritonX-100 in PBS for 5 min. After blocking with 5% bovine serum albumin at 37°C for 1 h, cells were incubated with primary antibodies at 4°C overnight. Cells were then incubated with Alexa Fluor-conjugated secondary antibodies for 2 h. After five additional washes with PBS, slides were mounted and analyzed by confocal microscopy (LSM880 META; Carl Zeiss MicroImaging) and Zeiss Zen Blue Edition Software (Carl Zeiss MicroImaging).

**RNA interference-mediated gene silencing**

The following sequences were used for RNA interference-mediated gene silencing in SH-SY5Y-APPswe cells: SIRT1-specific siRNA: 5'-UGAAGUGCCUCAGAUAUUA-3' RARβ-specific siRNA: 5'-CAGCUGAGUUGGACGAUCU-3', PGC-1α-specific siRNA: 5'-CCAAGACUCUAGACAACUA-3', COX IV-specific siRNA: 5'-CGCCATGCAACTCCATGCCTATTTA-3', and negative control siRNA: 5'-CGUCCUGACCUUUGAGUAUCU-3'. All siRNAs were synthesized by GenePharma (Shanghai, China). Cells were transfected specific siRNA oligonucleotides using Lipofectamine 3000 and assayed for gene silencing at 24 h post-transfection by Western blotting.

**Semi-quantitative RT-PCR**

PCR was performed as described previously . Total RNA (500 ng) was reverse transcribed using SuperScript III First-Strand Synthesis System (Invitrogen, 18080051) according to the manufacturer’s protocol. Primers for APP (forward primer: 5'-TGGAGGTACCCACTGATGGT-3', reverse primer: 5'-ACTGCATGTCTCTTTGGCGA-3'), ADAM10 (forward primer: 5'-CGGGGATGGGAGGTCAGTAT-3', reverse primer: 5'-AAATGTGCCACCACGAGTCT-3'), BACE1 (forward primer: 5'-CAGGCTTGTTCTTCACAGGG-3', reverse primer: 5'-ACCACAAAGCCTGGCAATCTC-3'), and GAPDH (forward primer: 5'-AGAAGGCTGGGGCTCATTTG-3', reverse primer: 5'-AGGGGCCATCCACAGTCTTC-3'). The amplified products were electrophoresed on a 1.5% agarose gel stained with GoldView dye and observed using the Quantity One software (Bio-Rad, Hercules, CA, USA).

**Detection of** **cellular ATP levels**

The levels of ATP in cells were determined using an ATP bioluminescence assay kit (Beyotime Institute of Biotechnology, Shanghai, China), according to the manufacturer’s instructions. Briefly, harvested cultured cells were lysed with a lysis buffer, followed by centrifugation at 12,000*g* for 5 min at 4°C. In 6-well plates, 50 μL of each supernatant was mixed with 100 μL of ATP detection working dilution. Finally, the levels of cellular ATP were determined by an Infinite 2000 plate reader (TECAN, Switzerland). Standard curves were also generated, and the protein concentration of each treatment group was determined using a Bradford protein assay.

**Measurement of intracellular cAMP levels**

After treatment with PBMT, total cell extracts from cultured cells were obtained immediately by lysing methods. After centrifugation to remove cell debris, we collected supernatants for cAMP measurement. The total amount of intracellular cAMP was determined according to the protocol provided with the cAMP immunoassay kit (R&D Systems, Minneapolis, MN, USA).

**Complexes I,** **II, III, IV and V activity assays**

After PBMT treatment, cells were harvested and lysed immediately with ice-cold lysate buffer and cell extracts were obtained. After centrifugation to remove cell debris, we collected the protein in the supernatants. The enzymatic activity of complex IV in cells was assessed by measuring cytochrome c oxidation using a COX activity testing kit (Genmed Scientifics Inc., Arlington, MA, USA). COX activity was determined by measuring the increase in absorbance at a wavelength of 550 nm using an Infinite 2000 plate reader. Complexes I, II, III, and V activities were assayed using the commercial activity detection kits (Cayman Chemical Co., USA) following the manufacturer’s recommendations.

**Evaluation of mitochondrial membrane potential** **(ΔΨmt)**

The mitochondrial membrane potential was determined using the fluorescent cationic dye, Rhodamine 123 (10 μM; Beyotime Institute of Biotechnology). Cells were allowed to reach exponential growth for 24 h before treatment. After PBMT treatment, the medium was removed, and the cells were incubated with Rhodamine 123 for 30 min at 37°C. Rhodamine 123 was taken up selectively by mitochondria in a ΔΨmt-dependent manner. Cells were washed three times with PBS, then observed and detected using confocal microscopy and flow cytometry (Becton Dickinson), respectively.

**Supplementary References**

Henderson TA, Morries LD (2015). Near-infrared photonic energy penetration: can infrared phototherapy effectively reach the human brain? *Neuropsychiatric disease and treatment*. 11, 2191-2208.

Liang J, Liu L, Xing D (2012). Photobiomodulation by low-power laser irradiation attenuates Abeta-induced cell apoptosis through the Akt/GSK3beta/beta-catenin pathway. *Free radical biology & medicine*. 53, 1459-1467.

Meng C, He Z, Xing D (2013). Low-level laser therapy rescues dendrite atrophy via upregulating BDNF expression: implications for Alzheimer's disease. *The Journal of neuroscience: the official journal of the Society for Neuroscience*. 33, 13505-13517.

Qin W, Yang T, Ho L, Zhao Z, Wang J, Chen L, Zhao W, Thiyagarajan M, MacGrogan D, Rodgers JT, Puigserver P, Sadoshima J, Deng H, Pedrini S, Gandy S, Sauve AA, Pasinetti GM (2006). Neuronal SIRT1 activation as a novel mechanism underlying the prevention of Alzheimer disease amyloid neuropathology by calorie restriction. *The Journal of biological chemistry*. 281, 21745-21754.

Trinchese F, Liu S, Battaglia F, Walter S, Mathews PM, Arancio O (2004). Progressive age-related development of Alzheimer-like pathology in APP/PS1 mice. *Annals of neurology*. 55, 801-814.

Zhang H, Wu S, Xing D (2012). Inhibition of Abeta(25-35)-induced cell apoptosis by low-power-laser-irradiation (LPLI) through promoting Akt-dependent YAP cytoplasmic translocation. *Cellular signalling*. 24, 224-232.

Zhang Z, Liu L, Wu S, Xing D (2016). Drp1, Mff, Fis1, and MiD51 are coordinated to mediate mitochondrial fission during UV irradiation-induced apoptosis. *FASEB journal: official publication of the Federation of American Societies for Experimental Biology*. 30, 466-476.
